# Associates of Insomnia in People with Chronic Spinal Pain: A Systematic Review and Meta-Analysis

**DOI:** 10.3390/jcm10143175

**Published:** 2021-07-19

**Authors:** Thomas Bilterys, Carolie Siffain, Ina De Maeyer, Eveline Van Looveren, Olivier Mairesse, Jo Nijs, Mira Meeus, Kelly Ickmans, Barbara Cagnie, Dorien Goubert, Lieven Danneels, Maarten Moens, Anneleen Malfliet

**Affiliations:** 1 Pain in Motion Research Group (PAIN), Department of Physiotherapy, Human Physiology and Anatomy, Faculty of Physical Education and Physiotherapy, Vrije Universiteit Brussel, 1090 Brussels, Belgium; thomas.bilterys@vub.be (T.B.); carolie18@hotmail.com (C.S.); Ina_dm@hotmail.com (I.D.M.); Eveline.VanLooveren@UGent.be (E.V.L.); jo.nijs@vub.be (J.N.); kelly.ickmans@vub.be (K.I.); 2 Department of Rehabilitation Sciences and Physiotherapy, Faculty of Medicine & Health Sciences, Ghent University, 9000 Ghent, Belgium; Mira.Meeus@UGent.be (M.M.); barbara.cagnie@ugent.be (B.C.); goubertdorien@gmail.com (D.G.); lieven.danneels@ugent.be (L.D.); 3 Pain in Motion International Research Group, 1090 Brussels, Belgium; 4 Experimental and Applied Psychology, Faculty of Psychology and Educational Sciences, Vrije Universiteit Brussel, 1050 Brussels, Belgium; olivier.mairesse@vub.be; 5 Sleep Laboratory and Unit for Chronobiology, Brugmann University Hospital, 1020 Brussels, Belgium; 6 Department of Physical Medicine and Physiotherapy, University Hospital Brussels, 1090 Brussels, Belgium; 7 Department of Rehabilitation Sciences and Physiotherapy (MOVANT), Faculty of Medicine and Health Sciences, University of Antwerp, 2610 Antwerpen, Belgium; 8 Research Foundation Flanders (FWO), 1000 Brussels, Belgium; 9 Department of Neurosurgery and Radiology, University Hospital Brussels, 1090 Brussels, Belgium; maarten.TA.moens@vub.be; 10 Center for Neuroscience, Vrije Universiteit Brussel, 1090 Brussels, Belgium

**Keywords:** back pain, neck pain, associates, socio-demographic factors, psychosocial factors, lifestyle factors, sleep–wake disorders, systematic review

## Abstract

Insomnia is a major problem in the chronic spinal pain (CSP) population and has a negative impact on health and well-being. While insomnia is commonly reported, underlying mechanisms explaining the relation between sleep and pain are still not fully understood. Additionally, no reviews regarding the prevention of insomnia and/or associated factors in people with CSP are currently available. To gain a better understanding of the occurrence of insomnia and associated factors in this population, we conducted a systematic review of the literature exploring associates for insomnia in people with CSP in PubMed, Web of Science and Embase. Three independent reviewers extracted the data and performed the quality assessment. A meta-analysis was conducted for every potential associate presented in at least two studies. A total of 13 studies were found eligible, which together identified 25 different potential associates of insomnia in 24,817 people with CSP. Twelve studies had a cross-sectional design. Moderate-quality evidence showed a significantly higher rate for insomnia when one of the following factors was present: high pain intensity, anxiety and depression. Low-quality evidence showed increased odds for insomnia when one of the following factors was present: female sex, performing no professional activities and physical/musculoskeletal comorbidities. Higher healthcare use was also significantly related to the presence of insomnia. One study showed a strong association between high levels of pain catastrophizing and insomnia in people with chronic neck pain. Last, reduced odds for insomnia were found in physically active people with chronic low back pain compared to inactive people with chronic low back pain. This review provides an overview of the available literature regarding potential associates of insomnia in people with CSP. Several significant associates of insomnia were identified. These findings can be helpful to gain a better understanding of the characteristics and potential origin of insomnia in people witch CSP, to identify people with CSP who are (less) likely to have insomnia and to determine directions of future research in this area.

## 1. Introduction

Chronic spinal pain (CSP) is a highly prevalent and debilitating condition associated with poor quality of life and high socioeconomic impact [1,2,3,4,5]. Furthermore, CSP can coexist with many comorbidities (like other chronic diseases), which generally leads to larger negative effects on physical and mental functioning, a reduced treatment response, higher levels of disability and higher costs compared to CSP alone [6,7,8].

Insomnia, defined as the presence of a long sleep latency, frequent nocturnal awakenings, prolonged periods of wakefulness during the sleep period or early awakenings, is common in people with CSP [9,10,11,12]. Up to 59% report insomnia, making it one of the most reported comorbidities in CSP [9,10,11,12]. Moreover, people with chronic low back pain are 18 times more likely to experience insomnia compared to people without chronic low back pain [11]. If left untreated, insomnia negatively impacts mood, physical symptoms, pain sensitivity, fatigue and health-related quality of life [13,14]. Additionally, insomnia is related to less productivity and increased work absenteeism [15]. Considering all of the above, co-occurring CSP and insomnia present a serious public health challenge which is currently rarely addressed in treatment [11].

Currently, underlying mechanisms explaining the relation between sleep and pain are still not fully understood [16]. A recent review provided an overview of the available evidence regarding investigated putative mediating variables on the pathway between sleep variables and pain intensity [17]. Based on the available body of research, they speculated that psychological and physiological components of emotional experience and attentional processes are likely mediators. However, this review focusses on the factors influencing the link between sleep and pain (i.e., mediators) in the general pain population. None of the included studies investigated mediators or associated factors specifically in people with CSP. Additionally, the review did not include studies which investigated potential associated factors if no formal test of mediation or a test of the significance of mediated effects was conducted.

A clear overview of factors (including socio-demographic, psycho-social and lifestyle factors) associated with insomnia in people with CSP could lead to a better understanding, a change in decision making and further improvement of preventive and treatment strategies (i.e., targeting possible identified factors). Yet, since such an overview is currently unavailable, the purpose of this systematic review and meta-analysis is to provide an overview of associates of insomnia in people with CSP. The primary aim of this review is to determine which factors are associated with insomnia in people with CSP. The secondary aim is to determine the strength of association for these factors.

## 2. Methods

This systematic review was conducted in accordance with the PRISMA guidelines and initially registered in the PROSPERO database (registry number CRD42018116710) [18]. A search for eligible studies was performed in three electronic databases, i.e., PubMed, Web of Science and Embase. The last search was conducted on 12 September 2019.

### 2.1. Identification and Selection of Studies

#### 2.1.1. Eligibility Criteria

Studies were eligible when meeting the following criteria: (1) including adults (>18 years) suffering from non-specific CSP (i.e., low back pain or neck pain not attributable to a specific pathology) for at least 3 months, (2) reporting insomnia-related outcomes [19,20], such as variables described in terms of sleep disturbances, sleep difficulties, sleep problems, restless sleep, disturbed sleep and sleep continuity;,(3) presenting data to identify associated factors with insomnia (i.e., odds ratios (ORs) or sufficient data to calculate the ORs) and (4) being written in English, French or Dutch.

The next criteria were applied for exclusion of studies: (1) abstracts, case reports, reviews, meta-analysis, letters and editorials, and (2) studies including participants diagnosed with specific medical conditions that can explain CSP (e.g., neck or back surgery in the past three years, osteoporotic vertebral fractures or rheumatologic diseases), diagnosed with chronic widespread pain (fibromyalgia or chronic fatigue syndrome), being shift workers, suffering from severe underlying sleep-related comorbidities or being pregnant or were pregnant in the preceding year.

#### 2.1.2. Information Sources

A systematic search was conducted in PubMed, Web of Science and Embase. The search in PubMed was performed using MeSH terms and free keywords based on the PECO-acronym, in which the “population” (P) was represented as people with CSP, the “exposure” (E) as potential associates and the “outcome” (O) as insomnia. Since studies without comparison groups were eligible, no search terms for “comparison group” (C) were used in the final search. Using free keywords, a comparable search was performed in Web of Science and Embase. No search filters were used. An overview of the applied search terms can be found in Table S1. Full search strategies of all databases are presented in Supplementary file S1. Additionally, reference lists of the relevant articles were hand-searched for additional eligible papers.

#### 2.1.3. Study Selection

After removing duplicates, three reviewers (C.S., I.D. and T.B.) independently screened all retrieved records to determine the eligibility. First, all records were screened by title and abstract in a blinded standardized manner using Rayyan software [21]. Studies that presented relevant data in accordance with the review question were included, even if the main research question was not relevant for this review. All discrepancies were resolved by consensus among the three researchers. When no agreement could be reached through discussion, a fourth author (A.M.) made the final decision. Reasons for exclusion were registered in all phases.

### 2.2. Data Collection Process

Three authors (C.S., I.D. and T.B) extracted the relevant data independently using a self-created data extraction form containing the following items: (1) author, (2) year of publication, (3) study design, (4) sample size, (5) nature of the sample, (6) age (years ± standard deviation), (7) assessment methods of insomnia, (8) prevalence rate of insomnia and (9) investigated or determinable potential associates. Data of factors/variables investigated in each study were extracted and presented in the tables, figures and meta-analyses of this review if ORs could be determined. Variables presented in the included studies without sufficient data to determine ORs were not included. One reviewer (T.B.) checked the extracted data and resolved any disagreement.

### 2.3. Risk of Bias Assessment of Individual Studies

Three reviewers (C.S., I.D. and T.B.) evaluated the methodological quality and risk of bias by using an adapted form of the Newcastle-Ottawa Scale (NOS), independently [22,23]. The NOS assesses the quality of studies in three main areas, i.e., selection, comparability and outcome or exposure, and leads to a maximum total score of 10. The quality of individual studies was rated as high, moderate and low based on designated thresholds [24]. Studies with a score of ≥7 out of 10 were considered high quality, studies with at least a score of 5 were rated as moderate quality and a score lower than 5 was considered low quality. Strict scoring criteria were determined a priori based on findings in the literature [25,26,27,28,29]. The response-rate was considered “satisfactory” when it reached ≥80% [25]. The sample size was considered “justified and satisfactory” if the number of needed participants was reached based on a sample size calculation, or when the study is a national or epidemiological study. For the section “comparability”, two points were possibly awarded: one for controlling for age or sex, and one for controlling for any other factor. Since age and sex differences in sleep are common [26,27,28,29], both factors were considered to be the most important factors to be controlled for. When an item was not described, a score of zero was given for that particular item. Overall risk of bias was considered “high” if the total score was 4 or lower. A score of at least 7 was considered as a “low” risk of bias. Uncertainties were solved by consensus among the three reviewers. The used NOS-version with details about the scoring criteria is provided in Supplementary file S2.

### 2.4. Summary Measures

The primary outcome measures were ORs with 95% confidence intervals (CIs). For every meta-analysis, a pooled OR (OR_p_) with 95% CI and *p*-value is presented. The statistical significance level (alpha) was set at 0.05.

### 2.5. Methods of Analysis

The number of subjects within the investigated subgroups (exposed subgroup and unexposed subgroup to the potential associated factor) with and without insomnia were collected to calculate ORs for each factor using Revman software (Review manager 5.3). Subsequently, random effects meta-analyses were performed for all the factors which were presented in at least two of the included studies [30]. The heterogeneity (I^2^) was assessed by the method proposed by Higgins et al. [30]. To determine the significance of the heterogeneity amongst studies, a Chi-squared (*X*^2^) test was conducted with an alpha set at 0.05 [31,32]. When a high heterogeneity (I^2^ > 50%) between studies was present [33], subgroup analyses (based on NOS-score, study design, pain location and used measurement tools) were performed to possibly clarify the underlying systematic differences and reduce the substantial heterogeneity.

### 2.6. Quality of Evidence

A modified version of the Grading of Recommendations Assessment, Development and Evaluation (GRADE) criteria was used to assess the quality of evidence for all analyses [34]. The criteria were modified to make them more suitable and relevant. The quality of evidence was downgraded from high by one level based on: phase of investigation (cross-sectional), study limitations (>25% of participants from studies with high risk of bias), inconsistency of results (I^2^ > 50%), imprecision (sample size < 400 participants), indirectness (e.g., inclusion of different populations and interventions) and publication bias (funnel plot and the Egger test if ≥10 studies [35]). Evidence was upgraded when there was at least a moderate effect size (OR > 2.5), or evidence of an exposure-response gradient.

## 3. Results

### 3.1. Study Selection

**Figure 1:**
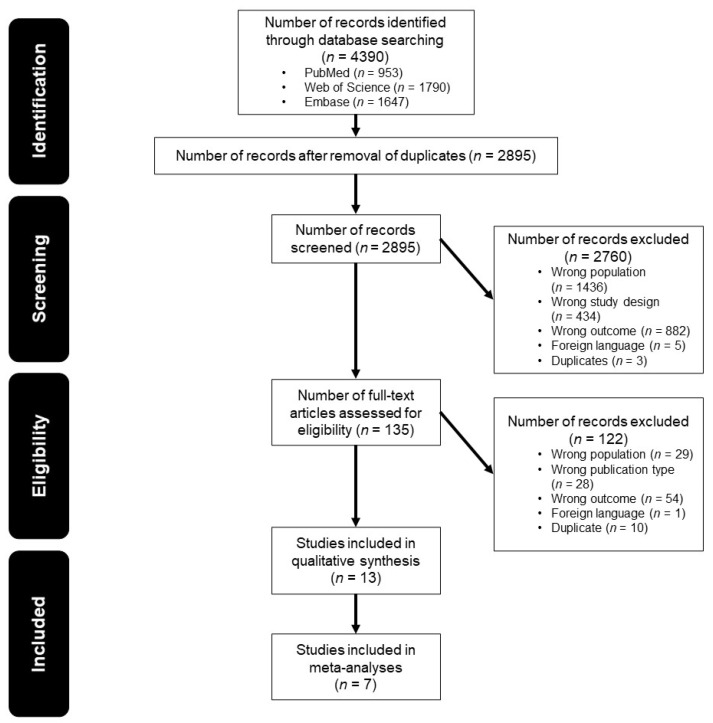
Flow diagram illustrating the study selection process.

The systematic search resulted in a total of 953 articles on PubMed, 1790 articles on Web of Science and 1647 articles on Embase. A total of 13 articles were included after the removal of duplicates, title and abstract screening and full-text eligibility assessment. No additional records were identified through hand-searching. The selection process is illustrated in Figure 1. An overview of the excluded articles assessed at full text and the reason for exclusion is presented in Table S2.

### 3.2. Study Characteristics

Twelve out of thirteen included studies were cross-sectional studies [11,12,36,37,38,39,40,41,42,43,44,45]. One included study was a cohort study [46]. A total of 24,817 participants were included across all studies, with sample sizes ranging from 70 to 10,849 participants [11,41]. The prevalence rate of insomnia across the studies ranged from 11% to 92% [12,38]. Nine studies used a validated questionnaire to retrieve information regarding the presence of insomnia [11,12,36,39,40,42,44,45,46]. Three other studies used a self-designed questionnaire [38,41,43] and one study made use of a health database [37]. A detailed overview of the characteristics of the included studies can be found in Table 1.

### 3.3. Risk of Bias within Studies

The overall methodological quality of the included studies is moderate to high, with scores ranging from 5 to 8 out of 10. Five out of thirteen studies were rated high quality, implying a “low” risk of bias. The other seven studies were rated as moderate quality, implying a “moderate” risk of bias. The main weakness was the relatively low response rate and the lack of comparison between the non-respondents and respondents (11 studies). The second most common source of bias was the lack of control for confounders (6 studies). The results of the quality assessment are presented in Table 2.

### 3.4. Synthesis of Results

In total, 25 different potential associates across 13 studies were identified. An overview of all included studies, including the identified factors and related ORs, is presented in Table 3. A meta-analysis was conducted for all the following factors which were presented in at least two of the included studies: sex (being female) [11,39,40,41,46], age (older age) [39,46], body mass index (BMI) [39,41], physical activity [36,41], professional activity [36,46], comorbidities [39,46], high pain intensity [39,45], depression [39,45] and anxiety [39,45]. No significant heterogeneity was found between studies analyzed for sex (I^2^ = 17%, *p* = 0.30), age (I^2^ = 0%, *p* = 0.99), BMI (I^2^ = 0%, *p* = 0.43), professional activity (I^2^ = 0%, *p* = 0.78), pain intensity (I^2^ = 0%, *p* = 0.92), depression (I^2^ = 0%, *p* = 0.98) and anxiety (I^2^ = 0%, *p* = 0.59). The assessment of the overall quality of the evidence for each analysis can be found in Table S3. Moderate-quality evidence was found for the factors pain intensity, anxiety and depression. Low- or very-low-quality evidence was found for the other examined factors.

### 3.5. Sex

**Figure 2:**
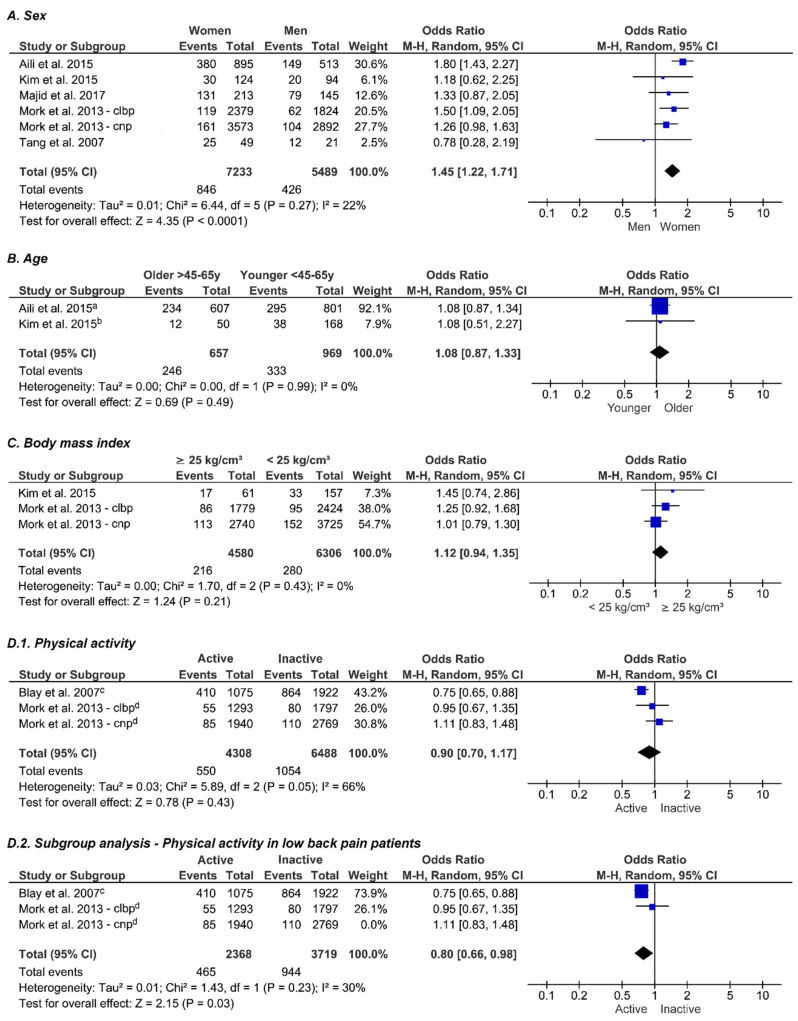

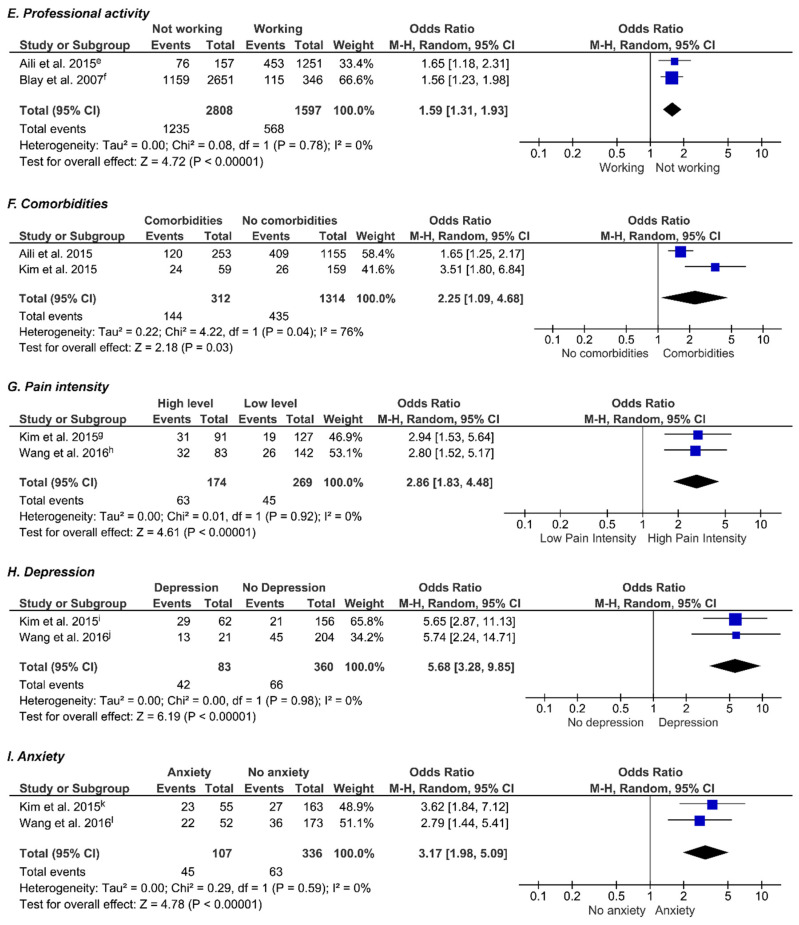
Forest plots showing odds ratios of several potential associated factors with insomnia in people with chronic spinal pain. A meta-analysis is conducted for the factors sex (*n* = 12,722), age *(n* = 1626), body mass index (*n* = 10,886), physical activity (*n* = 10,796), professional activity (*n* = 4405), comorbidities (*n* = 1626), pain intensity (*n* = 443), depression (*n* = 443) and anxiety (*n* = 443). Every blue box represents the observed odds ratio of the corresponding study. The size of every blue box is proportional to the weight of the study in the meta-analysis. The confidence intervals are represented by the horizontal lines through the blue boxes. The pooled odds ratio is represented by a black diamond, with the lateral tips of the diamond representing the associated confidence interval. Abbreviations: CNP, chronic neck pain; CLBP, chronic low back pain. ^a^ Aili et al. defined younger participants as people < 45 years [46]. ^b^ Kim et al. defined younger participants as people < 65 years [39]. ^c^ Blay et al. dichotomized physical activity in Yes/No but did not provide any detail about the level of physical activity used as a cut-off [36]. ^d^ Mork et al. defined physically active people as people performing more than one (accumulated) hour of exercise per week [41]. ^e^ Aili et al. defined performing no professional activity as “Unemployed for the last year/not working” [46]. ^f^ Blay at al. dichotomized professional activity as active/non-active but did not provide any further details [36]. ^g^ Kim et al. defined a high pain score as NRS ≥ 7 [39]. ^h^ Wang et al. defined a high pain score as VAS ≥ 7 [45]. ^i^ Kim et al. defined depression as a score of at least 8 on the HADS-D [39]. ^j^ Participants in the study of Wang et al. were screened by a board-certified psychiatrist for the presence of a current major depressive episode [45]. ^k^ Kim et al. defined depression as a score of at least 8 on the HADS-A [39]. ^l^ Participants in the study of Wang et al. were screened by a board-certified psychiatrist for the presence of any anxiety disorders [45].

Five studies reported on biological sex as a potential associated factor with insomnia (*n* = 12,722) [11,39,40,41,46]. The combined data indicates that female patients are more likely to have insomnia compared to male patients (OR_p_ 1.45, 95% CI = (1.22–1.71), *p* < 0.0001, low-quality evidence) (Figure 2A).

### 3.6. Age

Age was studied in 2 articles (*n* = 1626) [39,46]. No significant intergroup difference in insomnia prevalence was observed between older and younger people with CSP (OR_p_ 1.08, 95% CI = (0.87–1.33), *p* = 0.49, low-quality evidence) (Figure 2B).

### 3.7. Body Mass Index

Two studies reported on BMI (*n* = 10,886) [39,41]. No significant association was found between the presence of insomnia and a higher BMI (OR_p_ 1.12, 95% CI = (0.94–1.35), *p* = 0.21, low-quality evidence) (Figure 2C).

### 3.8. Physical Activity

Physical activity was studied in two studies (*n* = 10,796) [36,41]. No significant association was found between physical activity and the presence of insomnia in people with CSP (OR_p_ 0.90, 95% CI = (0.70–1.17), *p* = 0.43, very-low-quality evidence). A significant heterogeneity was observed (I^2^ = 66%, *p* = 0.43). Since Mork et al. examined chronic neck and back pain patients and reported both separately, a subgroup analysis including only the data regarding people with low back pain was performed [41]. This subgroup analysis resulted in an improvement of the heterogeneity (I^2^ = 30%, *p* = 0.23). Consequently, OR_p_ decreased to 0.80 (95% CI = (0.66–0.98), *p* = 0.03, low-quality evidence), indicating that insomnia is less common in physically active, chronic low back pain patients (Figure 2D).

### 3.9. Professional Activity

Two studies reported on professional activity (*n* = 4405) [36,46]. The pooled data showed that people with CSP without any professional activity are more likely to have insomnia compared to people with CSP who perform a job (OR_p_ 1.59, 95% CI = (1.31–1.93), *p* < 0.001, low-quality evidence) (Figure 2E).

### 3.10. Comorbidities

Physical or musculoskeletal comorbidities were studied in two studies (*n* = 1626) [39,46]. A significant intergroup difference (OR_p_ 2.25, 95% CI = (1.09–4.68), *p* = 0.03, very-low-quality evidence) with a significant heterogeneity (I^2^ = 76%, *p* = 0.04) was observed. Despite high heterogeneity, no subgroup analyses could be performed as comorbidities were only discussed in two articles. Furthermore, a subgroup analysis seems unnecessary due to the results of both studies being in the same direction (Figure 2F).

### 3.11. Pain Intensity

Pain intensity was considered as a putatively associated factor with insomnia in two studies (*n* = 443) [39,45]. The meta-analysis revealed that people with CSP with high pain intensity levels (VAS/NRS ≥ 7) are more likely to have insomnia compared to those with lower pain intensity levels (OR_p_ 2.86, 95% CI = (1.83–4.48), *p* < 0.001, moderate-quality evidence) (Figure 2G).

### 3.12. Depression

Two studies reported on depression as a factor (*n* = 443) [39,45]. The odds for insomnia were 5.68 times higher in people with CSP with depression compared to those without depression (OR_p_ 5.68, 95% CI = (3.28–9.85), *p* < 0.001, moderate-quality evidence) (Figure 2H).

### 3.13. Anxiety

Two studies discussed anxiety as a factor (*n* = 443) [39,45]. The pooled data demonstrated that people with CSP with anxiety are more likely to have insomnia compared to people with CSP without anxiety (OR_p_ 3.17, 95% CI = (1.98–5.09), *p* < 0.001, moderate-quality evidence) (Figure 2I).

### 3.14. Other

Each of the following factors were only discussed in one included article: income [36], medical consultation [36], hospitalization [36], self-rated health [36], prior opioid use [37], high C-reactive protein blood levels [38], pain duration [39], spine surgery history [39], shoulder/arm pain [39], neck mobility problems [39], myofascial pain [39], headache [39], use of sleep medication [14], pain catastrophizing [42], traumatic onset [43], healthcare use [44] and race [13]. A detailed overview of all included studies with the identified factors and their related ORs is presented in Table 3.

## 4. Discussion

The purpose of this systematic review and meta-analysis was to identify factors associated with the presence and development of insomnia in people with nonspecific CSP. A total of 13 studies were included, which together described 25 different potential associates of insomnia [11,12,36,37,38,39,40,41,42,43,44,45,46]. It was possible to carry out a meta-analysis for nine factors. Sex (being female), professional activity (not performing any professional activities), the presence of comorbidities, depression, anxiety and high pain intensity were significantly associated with elevated odds for insomnia. A significant heterogeneity was found for the factors of physical activity and comorbidities. A subgroup analysis was only possible for the factor physical activity, which became significant for people with chronic low back pain. Age and BMI could not be identified as associates.

Included studies looked into the possibility of the factors sex and age as associates of insomnia in people with CSP. The pooled data regarding sex as an associate showed that the odds for insomnia were 1.45 times higher for females compared to males (low-quality evidence). Similar results are found in the general population, with woman being almost 1.5 times more likely to develop insomnia compared to men [28]. It is suggested that this higher rate of insomnia in females might be explained by a higher prevalence of anxiety and depression, potentially indirectly induced by genetic factors [28]. However, underlying reasons for these sex differences still remain unclear since insomnia could not be solely explained by the higher prevalence of anxiety and depression alone. Different to the CSP population, age does appear to be associated with insomnia in the general population, with older adults showing a higher prevalence of insomnia [28,47]. As people get older, normal changes occur in our sleep architecture (e.g., more light sleep and fragmentation) [48]. However, these changes can contribute to the development of insomnia. Besides these natural changes of sleep, other comorbidities and specific sleep pathologies which can negatively influence sleep are also more common as people get older [49,50]. Furthermore, sleep difficulties in older adults seem to be more related to age-related conditions rather than to age itself [51,52]. Not finding this relation with age in people with CSP can be explained by the possible dominating influence of the characteristics of the pain condition. It is likely that pain is the predominant reason for insomnia in people with CSP, which could potentially overshadow or negate the effect of age on sleep. Another explanation might be the low number of included studies. Additional studies might increase the precision of the OR_p_. However, it is likely that age has a negligible influence on the presence of insomnia in CSP since the 95% CI is relatively small and the OR_p_ is very close to one. Yet, as age and sex are fixed factors, that cannot be targeted in therapy, focusing on other modifiable factors (such as comorbidities, pain intensity, depression and anxiety) seems more clinically relevant.

This systematic review with meta-analysis demonstrates that people with CSP with high pain severity (NRS/VAS ≥ 7) are almost 3 times more likely to have insomnia (moderate-quality evidence). However, since only 2 studies were included in the meta-analysis, some caution is warranted regarding the strength of the results. Nevertheless, the results are in accordance with the findings of a recent review investigating relationships, comorbidities and treatments in chronic pain and sleep disturbances, which indicated that sleep problems in people with chronic pain are associated with greater pain severity [53]. Evidence strongly suggests a bidirectional relationship, with pain and sleep co-existing and impacting each other [54,55]. Insomnia and pain seem to share similar pathways, such as mesolimbic dopaminergic pathways and serotoninergic pathways [16,56]. Generally, pain is associated with an increased stress-response and elevated levels of arousal [57], which can negatively affect sleep [58]. Furthermore, people with chronic pain are prone to start worrying about their health, which can further aggravate poor sleep [11,59,60,61]. Additionally, even a limited amount of sleep loss appears to have a de-activating effect on several analgesic systems, while activating hyperalgesic systems [16]. Furthermore, impaired sleep can result in low-grade inflammatory responses [62,63], which is found to potentially affect brain function [64] and increase pain sensitivity [63,65,66]. This bidirectional relationship creates a vicious cycle which can perpetuate and amplify sleep problems and pain (i.e., increasing pain disrupting the sleep and sleep disturbances exacerbating the pain). Taking all findings into account, the results of our analysis regarding pain intensity seems to be in line with the current research findings of the general chronic pain population, indicating that pain intensity has a clear impact on sleep. However, underlying mechanisms explaining the relation between sleep and pain are still not fully understood [16]. Addressing the vicious pain–sleep cycle in the evaluation and treatment of CSP seems to be essential to deliver the best possible care.

Similar to the link with pain intensity, the presence of depression and/or anxiety in CSP is linked to the prevalence of insomnia according to our results (moderate-quality evidence). However, since only two studies were included in the meta-analysis of both anxiety and depression, some caution is warranted regarding the strength of the results. Nevertheless, the strong associations of both factors do not come as a surprise since depression and anxiety are considered as the most prevalent comorbidities of both pain [67,68] and insomnia [69]. Furthermore, people with co-occurring pain and sleep problems appear to be more likely to present comorbid depression, catastrophizing, anxiety and suicidal ideation [53]. Moreover, previous research has demonstrated complex interactions between pain, sleep and depression, without a clear causal ordering [53,54]. Similarly, anxiety is found to be closely related with pain and insomnia, but the direction and underlying mechanisms of these relations are still unclear [68,70]. Given their relationship with pain and insomnia, addressing both depression and anxiety symptoms as an integral part of the evaluation and treatment of people with CSP and comorbid insomnia seems warranted.

Two studies looked at physical activity, which was found to be a non-significant associate after pooling (very-low-quality evidence). However, one could expect that inactivity would be an associate since there is sufficient evidence that physical activity has small but still positive effects on sleep in the general population [71]. Furthermore, physical activity has been identified as a strong “Zeitgeber” (i.e., a cue that helps to synchronize our biological rhythm to a 24 h cycle) [72]. Moreover, evidence shows that physical activity is beneficial, and therefore recommended, in people with CSP [73,74,75,76]. Importantly, our analysis showed that statistical heterogeneity was present, indicating a discrepancy between the data of both studies. After applying a subgroup analysis based on pain location, the heterogeneity improved, and physical activity became a small but significant protective factor for insomnia in people with chronic low back pain (low-quality evidence). This implies that physically active back pain patients are less likely to have insomnia.

A notable significant OR of 7.16 was found for pain catastrophizing, indicating that people with CSP with high levels of catastrophizing are much more likely to have insomnia [42]. However, pain catastrophizing was only investigated by one study, which only included people with chronic neck pain [42]. Therefore, the strength of the relation between insomnia and pain catastrophizing is rather indicative. It might be that studies that investigated anxiety and depression as factors considered catastrophizing as a part of the anxiety/depression complex since they share common elements and are closely related [77]. While there is some overlap with other cognitive and emotional processes, it is clear that catastrophizing is a unique construct [77]. Nevertheless, pain catastrophizing can be considered a clinically important psychological factor on its own given the high OR and its central role in the development of chronic disabling pain [42,78,79]. Therefore, targeting and reducing pain catastrophizing should be considered in CSP management.

Lastly, several studies investigating different aspects of healthcare use (i.e., medical consultations, number of hospitalizations, number of healthcare visits/year and opioid use) were included in this review [36,37,44]. Since each reported healthcare-related factor embodied a specific element of healthcare use and different thresholds for dichotomizations were used, the decision was made to not pool the data. However, all factors related to healthcare use show significantly higher odds (ranging from 1.45 to 2.96), indicating that people with CSP and comorbid insomnia are making significantly more use of the healthcare system compared to the average person with CSP.

Since the majority of chronic neck pain and chronic back pain (about 90%) can be considered non-specific/idiopathic [80,81], the investigated target population of this review were people with non-specific CSP. This implies that the presented results regarding several factors and their association with insomnia may vary in people with a specific diagnosis. However, a study by Kim et al. investigating risk factors for insomnia in a mixed sample of people with chronic low back pain with varying diagnoses (including lumbar disc herniation, spinal stenosis, spondylolisthesis, musculoskeletal back pain and mixed cases) showed similar results [82]. The study indicated that people with chronic low back pain with high pain intensity levels (VAS ≥ 7), comorbid musculoskeletal pain conditions and neuropathic pain components anxiety (HADS-A ≥ 8) and/or depression (HADS-D ≥ 8) were more likely to have insomnia (respectively 2.57, 14.71, 3.42, 3.14 and 5.58 times more likely), which is in accordance with the results of our review. In this study, sex, age and BMI were not identified as associates. However, a similar OR was found for sex (OR 1.40, 95% CI = (0.88–2.23)). A study of Yun et al. investigated associated factors with insomnia in a sample of 194 people diagnosed with failed back surgery syndrome [83]. Pain intensity (VAS ≥ 7), catastrophizing (≥30 PCS), anxiety (HADS-A ≥ 8) and depression (HADS-D ≥ 8) were found to be significantly related to insomnia. Compared to our results in people with non-specific CSP, higher ORs were found for all these factors in this sample of people with failed back surgery (respectively 5.01, 11.70, 8.09 and 9.53), suggesting an even stronger relation between these factors and insomnia in people diagnosed with failed back surgery syndrome. In contrast with our results, sex and comorbid musculoskeletal pain were not identified as risk factors. This suggests, despite some similarities, that associates and their strength of association with insomnia probably vary between non-specific CSP and CSP with a specific origin. Furthermore, associates might also vary between people with different CSP diagnoses. Nevertheless, the results of this review can serve as a basis since the majority of chronic low back and chronic neck pain is non-specific.

### 4.1. Strengths and Limitations

To our knowledge, this is the first systematic review with meta-analysis which provides a clear overview of associates of insomnia in people with CSP. This review has several strengths, including a rigorous methodology. First, this review was conducted in accordance with the PRISMA guidelines, which ensures a transparent, stepwise and complete approach. Second, we were able to perform several meta-analyses and one subgroup analysis which overcomes the issue of small sample sizes and makes it possible to draw more reliable and valid conclusions. Third, several comprehensive search strategies were used, including the screening of three different databases and additional hand-searching. Fourth, the screening and quality assessment has been conducted individually by three independent researchers. This improves the overall strength of the review by reducing the chance of making errors and missing an eligible study. Lastly, this review was a priori registered in the PROSPERO database, which avoids unplanned duplication, promotes transparency and reduces potential bias.

Despite the methodology used in this review and meta-analysis, a few limitations should be acknowledged. First, most included studies were cross-sectional in nature, implying that the results cannot provide information on causality, but rather provide an indication of association between the factors and insomnia. However, these ORs do indicate that insomnia is more prevalent in the presence of specific characteristics and can help to construct causal hypotheses. When translated to clinical practice, this means that the identified factors cannot predict whether a person with CSP will develop insomnia, yet they can help to identify those people with CSP that are very likely to suffer from insomnia. Second, most factors were only reported by less than four studies (except for sex). If more studies for each factor were available, the power and the generalizability of the meta-analyses would increase. According to recent research, five or more studies would be required to sufficiently power random-effects meta-analyses [84]. Despite the low number of studies for each factor, clear significant results were found for several factors. However, obtained results (i.e., ORs) would be a more precise representation if more studies were available. Additionally, more factors might become significant if more studies were available. However, most non-significant factors that potentially can become significant with increased number (and quality) of studies will be less relevant compared to the factors (with high ORs) that are clearly related to insomnia. Third, an adapted NOS for cross-sectional studies was used to assess the methodological quality, which was also applied for the included longitudinal cohort study [46], since no other valid alternative was available with the same point spread. However, the cohort study only measured sleep disturbances at baseline. Therefore, the extracted data to determine OR from this study could be considered as cross-sectional data. Last, the heterogeneity for the factors of “comorbidities” and “physical activity” was rather high. A possible explanation for this might be a different definition for physical activity and comorbidities, the use of different assessment methods and/or the use of different cut-off values. Regarding physical activity, for example, Blay et al. included people suffering from back pain who were aged 60 years or more, and used physical activity in a dichotomized manner (yes/no) [36]. On the other hand, Mork et al. focused on adults suffering from neck/shoulder and back pain, and classified participants as physically active when they performed more than one (accumulated) hour of exercise per week [41]. Furthermore, insomnia was also measured in different ways across all studies, which could have led to an increase in heterogeneity. Nevertheless, no significant heterogeneity was found in seven out of nine factors.

Taking these limitations into consideration, future studies should aim for large sample sizes and a rigorous methodology to ensure high-quality studies with strong and exact results. Furthermore, more factors that are targetable by different therapies (such as social-, psychological-, environmental-, contextual- and behavioral-related factors) should be investigated to make it possible to well-anticipate these associated factors and deliver the best possible care. Researchers should also implement a longitudinal design which makes it possible to draw conclusions regarding factors related to the development of insomnia in people with CSP. This would enable clinicians to make better predictions as to whether a patient with CSP is at risk of developing insomnia or not. Consequently, this will also help to develop preventive strategies or at least lead to early identification. Besides, future research should also focus on investigating and unravelling the underlying mechanisms explaining the relation between sleep and pain. This will help to gain a better understanding of the bidirectional relation and the underlying mechanisms. Complementary findings of future research regarding associated factors and underlying mechanisms can lead to an improvement of pharmacological and non-pharmacological approaches for the management of CSP comorbid with sleep disturbances and preventive strategies for insomnia.

### 4.2. Clinical Implications

While insomnia is a common and important issue in people with CSP, it is rarely addressed in the treatments for CSP. The results of this study can be helpful for clinicians to identify people with CSP early, who are very or less likely to have or develop insomnia based on the presence of several identified associated factors and the strength of the association. Based on the results, people with high pain intensity scores, who report depressive symptoms, who have anxiety and who catastrophize pain, have the highest chance of displaying insomnia. Furthermore, the identified associated factors might be a starting point to improve future treatment approaches. Nevertheless, more longitudinal research is needed to make firm conclusions regarding causality, the predictive value of the associated factors and the effectiveness of new treatment approaches, specifically targeting these associated factors.

This systematic review with meta-analysis shows that insomnia is relatively common in people with CSP. Several significant factors associated with insomnia in CSP were identified: moderate-quality evidence was found for the factors high pain intensity scores (NRS/VAS ≥ 7), depressive symptoms (HADS-D ≥ 8) and anxiety (HADS-A ≥ 8), and low-quality evidence was found for the factors female sex, the presence of comorbidities, performing no professional activities, pain catastrophizing and higher healthcare use. Low-quality evidence suggested that physically active low back pain patients are also less likely to suffer from insomnia. Having knowledge of these factors can help clinicians to identify patients who are (less) likely to have insomnia.

## Figures and Tables

**Table 1:** Characteristics of the included studies.

Author	Design	Sample Size (N)	Nature of the Sample	Age (Range and/or Years ± SD)	Pain Duration	Sleep Outcome	Prevalence Rates of Insomnia	Investigated Factors
Aili et al. 2015	C	1408	Care seeking CLBP and CNP, community sample	Range: 20–59 y	≥6 mo	Karolinska Sleep Questionnaire	NM	Sex, age, other physical illness, professional activity
Blay et al. 2007	CS	2997	CLBP, population-based sample	Range: 60–81 y	≥6 mo	Short Psychiatric Evaluation Schedule	42.5% sleep disturbance	Professional activity, income, medical consultation, hospitalizations, self-rated health, physical activity
Dimarco et al. 2018	CS	709	CLBP, sample in clinical setting	34.9 ± 11.9 y	Opioid naïve: 26.04 ± 50.21 mo Prior opioid users: 22.64 ± 46.26 mo	Data extracted from Military Health System Data Repository	19% insomnia	Prior opioid use
Ho et al. 2019	CS	6559	CLBP, community sample	52.2 ± 15.2 y Range: 19.1–95.9 y	≥3 mo	Modified insomnia criteria from DSM-5	10.9% insomnia	High CRP level
Kim et al. 2015	CS	218	CNP, sample in clinical setting	52.8 ± 14.3 y Range: 20–83 y	≥3 mo	Insomnia Severity Index	53.7% mild to severe insomnia	Sex, age, BMI, pain duration, pain score, spine surgery history, shoulder or arm pain, neck mobility problems, myofascial pain components, anxiety, depression, headache, comorbid musculoskeletal conditions
Majid et al. 2017	CS	358	CLBP, sample in clinical setting	NM	≥3 mo	Insomnia Severity Index	58.7% sleep disturbance	Sex
Marin et al. 2006	CS	268	CLBP, sample in clinical setting	47 y ± NM Range: 18–89 y	≥6 mo	Pittsburgh Sleep Quality Index	92% sleep disturbances	Sleep medication intake after pain
Mork et al. 2013	CS	10,849	CLBP and CNP, community sample	43.0 ± 13.9 y	≥3 mo	Self-Reported Questionnaire	NM	Sex, physical activity, BMI
Park et al. 2016	CS	256	CNP, sample in clinical setting	52.8 ± 14.7 y Range: 20–84 y	≥3 mo	Insomnia Severity Index	24.22% clinical insomnia	Pain catastrophizing
Ris et al. 2017	CS	200	CNP, sample in clinical setting	Traumatic: 43.5 ± 11.4 y Non-traumatic: 47.5 ± 11.3 y	≥6 mo	Self-reported Disturbed nights/week	19.5% sleep disturbances	Traumatic Onset
Shmagel et al. 2016	CS	700	CLBP, community sample	Range: 20–69 y	≥3 mo	NAHANS Questionnaires	52.7% sleep disturbances	Healthcare Use
Tang et al. 2007	CS	70	CLBP, sample in clinical setting	46 ± 10.9 y Range: 18–65 y	≥6 mo	Insomnia Severity Index	53% with moderate or severe insomnia	Sex, race
Wang et al. 2016	CS	225	CLBP	40.7 ± 11.4 y	≥3 mo	Insomnia Severity Index	25.8% clinical insomnia	Depression, anxiety, severity of CLBP

Abbreviations: BMI, body mass index; C, cohort; CLBP, chronic low back pain; CNP, chronic neck pain; CRP, C-reactive protein; CS, cross-sectional; DSM-5, the Diagnostic and Statistical Manual of Mental Disorders 5th Edition; mo, month; NHANES, National Health and Nutrition Examination Survey; NM, not mentioned; y, year.

**Table 2:** Quality assessment by the Adapted Newcastle–Ottawa scale.

Studies	Selection				Comparability	Outcome		Total
	Representativeness of the Sample (Maximum 1 star)	Sample Size (Maximum 1 Star)	Non-Respondents (Maximum 1 Star)	Ascertainment of the Exposure (Factor) (Maximum 2 Stars)	Confounding Factors (Maximum 2 Stars)	Assessment of the Outcome (Maximum 2 Stars)	Statistical Test (Maximum 1 Star)	Mean = 6.23 Median = 6
Aili et al. 2015	☆	☆		☆☆	☆☆	☆	☆	8
Blay et al. 2007	☆	☆	☆	☆		☆	☆	6
Dimarco et al. 2019	☆			☆	☆☆	☆☆	☆	7
Ho et al. 2019	☆	☆		☆☆	☆☆	☆	☆	8
Kim et al. 2015	☆			☆☆		☆	☆	5
Majid et al. 2017	☆	☆		☆☆		☆		5
Marin et al. 2006	☆	☆		☆		☆	☆	5
Mork et al. 2014	☆	☆		☆	☆	☆	☆	6
Park et al. 2016	☆	☆		☆☆		☆	☆	6
Ris et al. 2017	☆	☆		☆		☆	☆	5
Shmagel et al. 2016	☆	☆		☆	☆☆	☆	☆	7
Tang et al. 2007	☆			☆☆	☆	☆	☆	6
Wang et al. 2016	☆			☆☆	☆☆	☆	☆	7

The quality of the included studies is scored in three main areas, i.e., selection, comparability and outcome or exposure. Every star represents one point, which leads to a maximum total score of 10. The quality of individual studies were rated as high, moderate and low based on designated thresholds [24]. Studies with a score of ≥7 out of 10 were considered high quality. Studies with at least a score of 5 were rated as moderate quality studies. A score lower than 5 was considered low quality. Overall risk of bias was considered “high” if the total score was 4 or lower. A score of at least 7 was consider as “low” risk of bias.

**Table 3:** Overview of included studies with the potential associates and related odds ratios.

Author	Factor	Number of Participants with Insomnia (n)	Number of Participants without Insomnia (n)	Number of Participants in Reference and Investigated Subgroup (n)	(Adjusted) Odds Ratio [95% CI]
Aili et al. 2015	Sex	529	879		
	- Women	380	515	895	1.80 [1.43–2.27]
	- Men	149	364	513	1.0
	Age	529	879		
	- ≥45 years	234	373	607	1.08 [0.87–1.34]
	- <45 years	295	506	801	1.0
	Other physical illness	529	879		
	- Yes	120	133	253	1.65 [1.25–2.17]
	- No	409	746	1155	1.0
	Professional activity	529	879		
	- Not working	76	81	157	1.65 [1.18–2.31]
	- Working	453	798	1251	1.0
Blay et al. 2007	Professional activity	1274	1723		
	- Yes	115	231	346	1.0
	- No	1159	1492	2651	1.56 [1.23–1.98]
	Income	1274	1723		
	- High	312	631	943	0.56 [0.48–0.66]
	- Low	962	1092	2054	1.0
	Medical Consultation	1274	1723		
	- Yes	1041	1299	2340	1.46 [1.22–1.74]
	- No	233	424	657	1.0
	Hospitalizations	1274	1723		
	- >1	359	323	682	1.70 [1.43–2.02]
	- ≤1	915	1400	2315	1.0
	Self-rated health	1274	1723		
	- Impaired	1117	1170	2287	3.36 [2.77–4.09]
	- Not impaired	157	553	710	1.0
	Physical activity	1274	1723		
	- Yes	410	665	1075	0.75 [0.65–0.88]
	- No	864	1058	1922	1.0
Dimarco et al. 2018	Opioid user	112	592		
	- Yes	93	391	484	2.52 [1.49–4.24]
	- No	19	201	220	1.0
Ho et al. 2019	CRP Level	719	5840		
	- Elevated or very high	205	1390	1595	1.27 [1.07–1.52]
	- Very high	37	256	296	1.25 [0.88–1.79]
	- Elevated	168	1134	1302	1.28 [1.06–1.54]
	- Normal	514	4450	4964	1.0
Kim et al. 2015	Sex	50	168		
	- Women	30	94	124	1.18 [0.62–2.25]
	- Men	20	74	94	1.0
	Age	50	168		
	- ≥65 years	12	38	50	1.08 [0.51–2.27]
	- <65 years	38	130	168	1.0
	BMI	50	168		
	- ≥25 kg/m^2^	17	44	61	1.45 [0.74–2.86]
	- <25 kg/m^2^	33	124	157	1.0
	Pain duration	50	168		
	- ≥1 year	28	78	106	1.47 [0.78–2.77]
	- <1 year	22	90	112	1.0
	Pain score	50	168		
	- ≥7 NRS	31	60	91	2.94 [1.53–5.64]; Adj. 2.46 [1.12–5.40]
	- <7 NRS	19	108	127	1.0
	History of spine surgery	50	168		
	- Yes	7	15	22	1.74 [0.50–6.04]
	- No	43	153	196	1.0
	Shoulder or arm pain	50	168		
	- Yes	31	99	130	1.14 [0.60–2.18]
	- No	19	69	88	1.0
	Neck mobility problems	50	168		
	- Yes	13	43	56	1.02 [0.50–2.10]
	- No	37	125	162	1.0
	Comorbid musculoskeletal pain conditions	50	168		
	- Yes	24	35	59	3.51 [1.80–6.84]; Adj. 2.82 [1.22–6.54]
	- No	26	133	159	1.0
	Comorbid neuropathic pain component	50	168		
	- Yes	16	24	40	2.824 [1.354–5.887]
	- No	34	144	178	1.0
	Myofascial pain components	50	168		
	- Yes	20	50	70	1.57 [0.82–3.03]
	- No	30	118	148	1.0
	Anxiety	50	168		
	- HADS-A ≥ 8	23	32	55	3.62 [1.84–7.12]; Adj. 1.42 [0.58–3.48]
	- HADS-A < 8	27	136	163	1.0
	Depression	50	168		
	- HADS-D ≥ 8	29	33	62	5.65 [2.87–11.13]; Adj. 3.69 [1.57–8.67]
	- HADS-D < 8	21	135	156	1.0
	Headache	50	168		
	- Yes	13	35	48	1.34 [0.64–2.78]
	- No	37	133	170	1.0
Majid et al. 2017	Sex	210	148		
	- Women	131	82	213	1.33 [0.87–2.05]
	- Men	79	66	145	1.0
Marin et al. 2006	Sleep medication intake after pain	230	18		
	- Yes	130	4	134	4.55 [1.45–14.25]
	- No	100	14	114	1.0
Mork et al. 2013	Sex				
	Low back pain	181	4203		
	- Women	119	2260	2379	1.50 [1.09–2.05]
	- Men	62	1762	1824	1.0
	Neck pain	265	6200		
	- Women	161	3412	3573	1.26 [0.98–1.63]
	- Men	104	2788	2892	1.0
	Activity Level: leisure time physical exercise				
	Low back pain	135	2955		
	- Inactive	80	1717	1797	1.0
	- Active	55	1238	1293	0.95 [0.67–1.35]
	Neck pain	195	4514		
	- Inactive	110	2659	2769	1.0
	- Active	85	1855	1940	1.11 [0.83–1.48]
	BMI				
	Low back pain	181	4022		
	- ≥25 kg/cm^3^	86	1693	1779	1.25 [0.92–1.68]
	- <25 kg/cm^3^	95	2329	2424	1.0
	Neck pain	265	6200		
	- ≥25 kg/cm^3^	113	2627	2740	1.01 [0.79–1.30]
	- <25 kg/cm^3^	152	3573	3725	1.0
Park et al. 2016	Pain catastrophizing	62	194		
	- High	42	44	86	7.16 [3.81–13.43]
	- Low	20	150	170	1.0
Ris et al. 2017	Traumatic onset	39	161		
	- Yes	19	101	120	0.56 [0.28–1.14]
	- No	20	60	80	1.0
Shmagel et al. 2016	Healthcare use	172	528		
	- ≥10 healthcare visits/year	124	246	370	2.96 [2.03–4.31]
	- <10 visits/year	48	282	330	1.0
Tang et al. 2007	Sex	37	33		
	- Women	25	24	49	0.78 [0.28–2.19]
	- Men	12	9	21	1.0
	Race	37	33		
	- Caucasian	26	20	46	1.54 [0.57–4.14]
	- Non-Caucasian	11	13	24	1.0
Wang et al. 2016	Depression (Diagnosis of major depressive episode)	58	167		
	- Yes	13	8	21	5.74 [2.24–14.71]
	- No	45	159	204	1.0
	Anxiety (Diagnosis of an anxiety disorder)	58	167		
	- Yes	22	30	52	2.79 [1.44–5.41]
	- No	36	137	173	1.0
	Pain score	58	167		
	- VAS ≥ 7	32	51	83	2.80 [1.52–5.17]
	- VAS < 7	26	116	142	1.0

Abbreviations: Adj., adjusted; BMI, body mass index; CI, confidence interval; HADS, Health Anxiety and Depression Scale; LBP, low back pain; PE, patients exposed; VAS, Visual Analogue Scale.

## Data Availability

Not applicable.

## Supplementary Materials

The following are available online at https://www.mdpi.com/article/10.3390/jcm10143175/s1, Supplementary file S1, Supplementary file S2, Table S1: Search Terms, Table S2: Overview excluded articles screened on full text, Table S3: Quality of Evidence - Modified version of the Grading of Recommendations Assessment, Development, and Evaluation criteria.
