# Supplementary material for: Associates of Insomnia in People with Chronic Spinal Pain: A Systematic Review and Meta-Analysis
Source: J Clin Med. 2021 Jul 19;10(14):3175. doi: 10.3390/jcm10143175 (PMC8304652; doi:10.3390/jcm10143175)
Supplement: Supplementary file 1 [file jcm-10-03175-s001.zip › Supplementary file S1 Searches databases.pdf]

Supplementary file S1. *Full search strategies of all used databases*

Search Query Pubmed

("Back Pain"[Mesh] OR "Neck Pain"[Mesh] OR back pain* OR neck pain* OR spine pain* OR spinal pain* OR Vertebrogenic pain* OR lumbar pain* OR cervical pain* OR Discogenic pain* OR facet pain* OR facet joint pain* OR zygapophyseal pain* OR zygapophyseal joint pain* OR Backache* OR discopathy OR lumbago OR whiplash OR trapezius myalgia OR dorsalgi* OR vertebral pain*) AND ("Sleep"[Mesh] OR "Sleep Wake Disorders"[Mesh] OR sleep problem* OR sleeping problem* OR sleep disorder* OR sleeping disorder* OR sleeping difficult* OR sleep difficult* OR sleep disturb* OR sleeping disturb* OR sleep pattern* OR sleeping pattern* OR sleepless* OR wakeful* OR sleep dysfunction* OR sleeping dysfunction* OR Non-restorative sleep* OR unrefreshing sleep* OR insufficient sleep* OR short sleep* OR sleep disease* OR sleeping sickness* OR sleep sickness* OR insomnia* OR "sleep quality" OR "sleep onset" OR sleep efficien* OR sleep fragment* OR sleep latency OR polysomnography OR sleep diar* OR actigraph* OR sleeping disease* OR "sleep continuity" OR dyssomni* OR sleep deprivat* OR hyposomni*) AND ("Epidemiology"[Mesh] OR "Epidemiologic Factors"[Mesh] OR "Prognosis"[Mesh] OR "etiology"[Subheading] OR risks OR etiology OR causalit* OR variable* OR indicator* OR incidence OR frequenc* OR causation* OR prognos* OR outcome* OR factor* OR predict* OR probabilit* OR prevalence* OR hazard ratio* OR odds ratio* OR determinant* OR contribut* OR characteristic* OR predispos* OR epidemiology OR cause*)

Search Query Embase

("back pain$":ti,ab,kw OR "neck pain$":ti,ab,kw OR "spine pain$":ti,ab,kw OR "spinal pain$":ti,ab,kw OR "vertebrogenic pain$":ti,ab,kw OR "lumbar pain$":ti,ab,kw OR "cervical pain$":ti,ab,kw OR "discogenic pain$":ti,ab,kw OR "facet pain$":ti,ab,kw OR "facet joint pain$":ti,ab,kw OR "zygapophyseal pain$":ti,ab,kw OR "zygapophyseal joint pain$":ti,ab,kw OR 'backache$':ti,ab,kw OR 'discopathy':ti,ab,kw OR 'lumbago':ti,ab,kw OR 'whiplash':ti,ab,kw OR 'trapezius myalgia':ti,ab,kw OR 'dorsalgi*':ti,ab,kw OR "vertebral pain$":ti,ab,kw) AND (sleep*:ti,ab,kw OR wakeful*:ti,ab,kw OR insomnia*:ti,ab,kw OR polysomnography:ti,ab,kw OR actigraph*:ti,ab,kw OR dyssomni*:ti,ab,kw OR hyposomni*:ti,ab,kw) AND (epidemiolog*:ab,kw,ti OR etiology:ab,kw,ti OR risk$:ab,kw,ti OR variable$:ab,kw,ti OR indicator$:ab,kw,ti OR incidence:ab,kw,ti OR frequenc*:ab,kw,ti OR prognos*:ab,kw,ti OR causation$:ab,kw,ti OR cause$:ab,kw,ti OR causalit*:ab,kw,ti OR outcome$:ab,kw,ti OR factor$:ab,kw,ti OR predict*:ab,kw,ti OR probabilit*:ab,kw,ti OR prevalence$:ab,kw,ti OR "hazard ratio$":ab,kw,ti OR "odds ratio$":ab,kw,ti OR determinant$:ab,kw,ti OR contribut*:ab,kw,ti OR predispos*:ab,kw,ti OR characteristic$:ab,kw,ti)

Search Query Web of Science

1
TOPIC: ("back pain$" OR "neck pain$" OR "spine pain$" OR "spinal pain$" OR "Vertebrogenic pain$" OR "lumbar pain$" OR "cervical pain$" OR "discogenic pain$" OR "facet pain$" OR "facet joint pain$" OR "zygapophyseal pain$" OR "zygapophyseal joint pain$" OR backache$ OR discopathy OR lumbago OR whiplash OR "trapezius myalgia"

OR dorsalgi* OR "vertebral pain$")
Indexes=SCI-EXPANDED, SSCI, A&HCI, CPCI-S, CPCI-SSH, ESCI Timespan=All years

2
TOPIC: (sleep* OR sleepless* OR wakeful* OR insomnia* OR polysomnography OR actigraph* OR dyssomni* OR hyposomni*)
Indexes=SCI-EXPANDED, SSCI, A&HCI, CPCI-S, CPCI-SSH, ESCI Timespan=All years

3
TOPIC: (epidemiolog* OR etiology OR risk$ OR variable$ OR indicator$ OR incidence OR frequenc* OR prognos* OR causation$ OR cause$ OR causalit* OR outcome$ OR factor$ OR predict* OR probabilit* OR prevalence$ OR "hazard ratio$" OR "odds ratio$" OR determinant$ OR contribut* OR predispos* OR characteristic$)
Indexes=SCI-EXPANDED, SSCI, A&HCI, CPCI-S, CPCI-SSH, ESCI Timespan=All years

4
#1 AND #2 AND #3
