# Supplementary material for: Associates of Insomnia in People with Chronic Spinal Pain: A Systematic Review and Meta-Analysis
Source: J Clin Med. 2021 Jul 19;10(14):3175. doi: 10.3390/jcm10143175 (PMC8304652; doi:10.3390/jcm10143175)
Supplement: Supplementary file 1 [file jcm-10-03175-s001.zip › Supplementary file S2.pdf]

Supplementary file S2 *Newcastle – Ottowa scale adapted for cross-sectional studies, based on the version of Modesti et al. 2016 [23]*

Selection

1) Representativeness of the sample:
    a) Truly representative of the average in the target population. * (all subjects or random sampling)
    b) Somewhat representative of the average in the target population. * (non-random sampling)
    c) Selected group of users.
    d) No description of the sampling strategy.
2) Sample size:
    a) Justified and satisfactory. *
    b) Not justified.
3) Non-respondents:
    a) Comparability between respondents and non-respondents characteristics is established, and the response rate is satisfactory. *
    b) The response rate is unsatisfactory, or the comparability between respondents and non-respondents is unsatisfactory.
    c) No description of the response rate or the characteristics of the responders and the non-responders.
4) Ascertainment of the exposure (risk factor):
    a) Validated measurement tool. **
    b) Non-validated measurement tool, but the tool is available or described.*
    c) No description of the measurement tool.

Comparability: (Maximum 2 stars)

1) The subjects in different outcome groups are comparable, based on the study design or analysis. Confounding factors are controlled.
    a) The study controls for sex or age. *
    b) The study control for any additional factor. *

Outcome: (Maximum 3 stars)

1) Assessment of the outcome:
    a) Independent blind assessment. **
    b) Record linkage. **
    c) Self-report. *
    d) No description.
2) Statistical test:
    a) The statistical test used to analyze the data is clearly described and appropriate, and the measurement of the association is presented, including confidence intervals and the probability level (p value). *
    b) The statistical test is not appropriate, not described or incomplete.

Strict scoring criteria were determined a priori based on findings in literature. The response-rate was considered "satisfactory" when it reached ≥80%[25]. The sample size was considered “justified and satisfactory” if the number of needed participants was reached based on a sample size calculation, or when the study is a national or epidemiological study. For the section “comparability”, two points were possibly awarded: one for controlling for age or sex, and one for controlling for any other factor. Since age- and sex differences in sleep are common[26-29], both factors were considered to be the most important factors to be controlled for. When an item was not described, a score of zero was given for that particular item.
