# Supplementary material for: Associates of Insomnia in People with Chronic Spinal Pain: A Systematic Review and Meta-Analysis
Source: J Clin Med. 2021 Jul 19;10(14):3175. doi: 10.3390/jcm10143175 (PMC8304652; doi:10.3390/jcm10143175)
Supplement: Supplementary file 1 [file jcm-10-03175-s001.zip › Table S1.pdf]

***Table S1** Search Terms*

	P<br>**Spinal Pain**	E<br>**Insomnia**	O<br>**Determinants**
MeSH terms	Back Pain<br>Neck Pain	Sleep<br>Sleep Wake Disorders	Epidemiology<br>Epidemiologic Factors<br>Prognosis<br>Etiology [subheading]
Free terms	Back pain*<br>neck pain*<br>spine pain*<br>spinal pain*<br>Vertebrogenic pain*<br>lumbar pain*<br>cervical pain*<br>Discogenic pain*<br>facet pain*<br>facet joint pain*<br>zygapophyseal pain*<br>zygapophyseal joint pain*<br>Backache*<br>discopathy<br>lumbago<br>whiplash<br>trapezius myalgia<br>dorsalgi*<br>vertebral pain*	sleep problem*<br>sleeping problem*<br>sleep disorder*<br>sleeping disorder*<br>sleeping difficult*<br>sleep difficult*<br>sleep disturb*<br>sleeping disturb*<br>sleep pattern*<br>sleeping pattern*<br>sleepless*<br>wakeful*<br>sleep dysfunction*<br>sleeping dysfunction*<br>Non-restorative sleep*<br>unrefreshing sleep*<br>Insufficient sleep*<br>short sleep*<br>sleep disease*<br>sleeping sickness*<br>sleep sickness*<br>insomnia*<br>"sleep quality"<br>"sleep onset"<br>sleep efficien*<br>sleep fragment*<br>sleep latency<br>polysomnography<br>sleep diar*<br>actigraph*<br>sleeping disease*<br>"sleep continuity"<br>dyssomni*<br>sleep deprivat*<br>hyposomni*	Risks<br>etiology<br>causalit*<br>variable*<br>indicator*<br>incidence<br>frequenc*<br>causation*<br>prognos*<br>outcome*<br>factor*<br>predict*<br>probabilit*<br>prevalence*<br>hazard ratio*<br>odds ratio*<br>determinant*<br>contribut*<br>characteristic*<br>predispos*<br>epidemiology<br>cause*
