# Supplementary material for: Associates of Insomnia in People with Chronic Spinal Pain: A Systematic Review and Meta-Analysis
Source: J Clin Med. 2021 Jul 19;10(14):3175. doi: 10.3390/jcm10143175 (PMC8304652; doi:10.3390/jcm10143175)
Supplement: Supplementary file 1 [file jcm-10-03175-s001.zip › Table S2.pdf]

Table S2 *Overview excluded articles screened on full text*

Number	Reference excluded article	Reason for exclusion
1	Abbasi M, Yazdi Z, Mahdavifar MM. Prevalence of musculoskeletal disorders in firefighters and its association with insomnia. Annals of the Rheumatic Diseases 2016; 75(0):1193.	Wrong publication type: Abstract conference paper
2	Alfoldi P, Wiklund T, Gerdle B. Comorbid insomnia in patients with chronic pain: a study based on the Swedish quality registry for pain rehabilitation (SQRP). Disability and Rehabilitation 2014; 36(20):1661-1669.	Wrong population: General chronic pain population
3	Alsaadi SM, McAuley JH,; Hush JM, Maher CG. Prevalence of sleep disturbance in patients with low back pain. Eur Spine J 2011; 20(5):737-743	Wrong publication type: Review. Data extracted from studies previously conducted by the authors or their colleagues between 2001 and 2009.
4	Alsaadi SM, McAuley JH,; Hush JM, Maher CG. Prevalence of sleep disturbance in patients with low back pain. Eur Spine J 2011; 20(5):737-743	Duplicate
5	Alsaadi SM, McAuley JH,; Hush JM, Maher CG. Erratum: Prevalence of sleep disturbance in patients with low back pain. Eur Spine J 2012; 21(3):554-560	Wrong publication type: Erratum.
6	Alsaadi SM, McAuley JH, Hush JM, Bartlett DJ, Henschke N, Grunstein RR, Maher CG. Detecting insomnia in patients with low back pain: accuracy of four self-report sleep measures. BMC Musculoskelet Disord 2013; 14(0):196	Wrong outcome: No risk factors for insomnia are presented and there is insufficient data to calculate the odds ratios.
7	Alsaadi SM, McAuley JH, Hush JM, Bartlett DJ, McKeough ZM, Grunstein RR, Dungan GC, Maher CG. Assessing sleep disturbance in low back pain: the validity of portable instruments. PLoS One 2014; 9(4):e95824	Wrong outcome: No risk factors for insomnia are presented and there is insufficient data to calculate the odds ratios.
8	Alsaadi SM, McAuley JH, Hush JM, Lo S, Bartlett DJ, Grunstein RR, Maher CG. The bidirectional relationship between pain intensity and sleep disturbance/quality in patients with low back pain. Clin J Pain 2014; 30(9):755-765	Wrong population: Mixed group of acute and chronic low back pain.
9	Amelot A, Mathon B, Haddad R, Renault MC, Duguet A, Steichen O. Low Back Pain Among Medical Students: A Burden and an Impact to Consider! Spine (Phila Pa 1976) 2019; 44(19):1390-1395.	Wrong outcome: No risk factors for insomnia are presented and there is insufficient data to calculate the odds ratios.
10	Artner J, Cakir B, Spiekermann J, Kurz S, Leucht F, Reichel H, Lattig F. Prevalence of	Wrong population: Inclusion of low back pain patients with

	sleep deprivation in patients with chronic neck and back pain: a retrospective evaluation of 1016 patients. J of Pain Res. 2013; 6(0):1-6.	specific diagnoses (e.g. intervertebral disc disease (osteochondritis, cervical, or lumbar disc herniation), spinal canal stenosis, spondylolisthesis, osteoporotic fracture (chronic)).
11	Asih S, Neblett R, Mayer TG, Gatchel RJ. Does patient-reported insomnia improve in response to interdisciplinary functional restoration for chronic disabling occupational musculoskeletal disorders? Spine (Phila Pa 1976) 2014; 39(17):1384-1392.	Wrong population: Mixed sample of patients with chronic pain including the following diagnoses: sprains, strains, contusions, fractures, dislocations, nerve, and soft-tissue injuries.
12	Asih S, Neblett R, Mayer TG, Brede E, Gatchel RJ. Insomnia in a chronic musculoskeletal pain with disability population is independent of pain and depression. Spine Journal 2014; 14(9):2000-2007.	Wrong population: Mixed chronic musculoskeletal pain population with several areas of injury.
13	Axen I. Pain-related Sleep Disturbance: A Prospective Study With Repeated Measures. Clin J Pain 2016; 32(3):254-259.	Wrong population: Mixed group of acute and persistent low back pain.
14	Axen I, Kwak L, Hagberg J, Jensen I. Does physical activity buffer insomnia due to back and neck pain? PLoS ONE 2017; 12(9).	Wrong population: Mixed population of workers with no, acute or chronic low back and/or neck pain in private companies. No subdivision was made for “no pain”, “acute pain” nor “chronic pain”. Several questions concerning musculoskeletal pain were used to create “pain risk profiles”, which were further used to investigate whether physical activity does buffer insomnia.
15	Bahouq H, Rkain H, Allali F, Hajjaj Hassouni N. Prevalence and severity of insomnia in chronic low back pain patients. Annals of the Rheumatic Disease 2013, 71:617.	Wrong publication type: Scientific abstract, poster presentations
16	Bahouq H, Allali F, Rkain H, Hmamouchi I, Hajjaj-Hassouni N. Prevalence and severity of insomnia in chronic low back pain patients. Rheumatol Int 2013; 33(5):1277-1281.	Wrong population: Inclusion of low back pain patients with specific diagnoses (e.g. lumbar disk herniation, spondylolisthesis, spinal stenosis).
17	Bailey N, Macleod J. Is patient age a predictive factor of likely outcome following	Wrong publication type: Abstract, conference paper.

	lumbar facet denervation procedure, in the management of chronic low back pain (CLBP)? Br. J. Pain 2019; 13:14	
18	Benedict TM, Singleton MD, Nitz AJ, Shing TL, Kardouni JR. Effect of Chronic Low Back Pain and Post-Traumatic Stress Disorder on the Risk for Separation From the US Army. Mil Med. 2019; 184(9-10):431-439.	Wrong outcome: No risk factors for insomnia are presented and there is insufficient data to calculate the odds ratios.
19	Bissell D, Ziadni M, Sturgeon J, Martin K, Guck A, Trost Z. The impact of perceived discrimination, injustice beliefs, and sleep disturbance on anger experience in chronic low back pain. Journal of Pain 2017; 18(4):S64.	Wrong publication type: Abstract conference paper
20	Blanco CMY, Delgado FE, Santos BR, Ugalde UL, Sánchez CB, Zubeldia GMA, Moratinos RAB. The pilates therapeutic method improves mood, well-being and quality of sleep in patients with chronic spinal pain. J. Physiol. Biochem. 2018; 74:(S52)	Wrong publication type: Abstract, conference paper
21	Bulthuis Y, Vollenbroek-Hutten M, Hermens H, Vendrig L, van Lummel R. Psychological distress, disturbed sleep and physical activity during the night in chronic low-back pain patients. Journal of Back and Musculoskeletal Rehabilitation 2004; 17(2):69-76.	Wrong outcome: No risk factors for insomnia are presented and there is insufficient data to calculate the odds ratios.
22	Burgess HJ, Burns JW, Buvanendran A, Gupta R, Chont M, Kennedy M, Bruehl S. Associations Between Sleep Disturbance and Chronic Pain Intensity and Function: A Test of Direct and Indirect Pathways. Clin J Pain. 2019; 35(7):569-576.	Wrong outcome: No risk factors for insomnia are presented and there is insufficient data to calculate the odds ratios.
23	Burgess HJ, Rizvydeen M, Kimura M, Pollack MH, Hobfoll SE, Rajan KB, Burns, JW. An Open Trial of Morning Bright Light Treatment Among US Military Veterans with Chronic Low Back Pain: A Pilot Study. Pain Med. 2019; 20(4):770-778.	Wrong outcome: No risk factors for insomnia are presented and there is insufficient data to calculate the odds ratios.
24	Chen Q, Hayman LL, Shmerling RH, Bean JF, Leveille SG. Characteristics of chronic pain associated with sleep difficulty in older adults: the Maintenance of Balance, Independent Living, Intellect, and Zest in the Elderly (MOBILIZE) Boston study. J Am Geriatr Soc 2011; 59(8):1385-1392.	Wrong population: Older, general chronic pain population.
25	Choi YS, Kim DJ, Lee KY, Park YS, Cho KJ, Lee JH, Rhim HY, Shin BJ. How does chronic back pain influence quality of life in	Wrong population: Inclusion of low back pain patients with specific diagnoses (e.g. lumbar disk herniation,

	koreans: a cross-sectional study. Asian Spine J 2014; 8(3):346-352.	spondylolisthesis, spinal stenosis).
26	Choudhary S, Hussain ME, Moscovitch A, Pandi-Perumal SR, Bahammam AS. Multimodal Physiotherapy Improves Pain, Functional Disability, Sleep Quality and Health Related Quality of Life in Chronic Mechanical Neck Pain Patients. International Journal of Health Sciences & Research. 2018; 8(3): 138-148.	Wrong outcome: No risk factors for insomnia are presented and there is insufficient data to calculate the odds ratios.
27	Costa N, Hodges PW, Ferreira ML, Makovey J, Setchell J. What Triggers an LBP Flare? A Content Analysis of Individuals' Perspectives. Pain Med. 2020; 21(1):13-20.	Wrong population: Inclusion of patients with acute and chronic low back pain. There was no exclusion based on low back pain duration, coexisting pain and comorbidities.
28	Cunningham JM, Blake C, Power CK, O'Keeffe D, Kelly V, Horan S, Spencer O, Fullen BM. The impact on sleep of a multidisciplinary cognitive behavioural pain management programme: a pilot study. Bmc Musculoskelet Disord. 2011; 12:5.	Wrong publication type: Protocol.
29	De Jaeger M, Goudman L, De Groote S, Rigoard P, Monlezun O, Moens M. Does Spinal Cord Stimulation Really Influence Sleep? Neuromodulation 2019; 22(3):311-316.	Wrong population: Patients diagnosed with failed back surgery syndrome (receiving spinal cord stimulation during the trail).
30	Doualla M, Aminde J, Aminde LN, Lekpa FK, Kwedi FM, Yenshu EV, Chichom AM. Factors influencing disability in patients with chronic low back pain attending a tertiary hospital in sub-Saharan Africa. BMC Musculoskelet Disord. 2019; 20: 25.	Wrong population: Inclusion of low back pain patients with specific diagnoses (e.g. disc desease and spinal stenosis).
31	Doualla M, Aminde J, Aminde LN, Lekpa FK, Kwedi FM, Yenshu EV, Chichom AM. Factors influencing disability in patients with chronic low back pain attending a tertiary hospital in sub-Saharan Africa. BMC Musculoskelet Disord. 2019; 20: 25.	Duplicate
32	Eadie J, van de Water AT, Lonsdale C, Tully MA, van Mechelen W, Boreham CA, Daly L, McDonough SM, Hurley DA. Physiotherapy for sleep disturbance in people with chronic low back pain: results of a feasibility randomized controlled trial. Arch Phys Med Rehabil. 2013; 94(11):2083-2092.	Wrong outcome: No risk factors for insomnia are presented and there is insufficient data to calculate the odds ratios.
33	Eadie J, Van Der Water A, Tully M, Mechelen W; Boreham C, McDonnagh S, Lonsdale C, Daly L, Hurley D. The	Wrong publication type: Abstract, conference paper.

	effectiveness of a walking programme, supervised exercise programme and usual physiotherapy on sleep disturbance in chronic low back pain: 3-month results of a feasibility randomized controlled trial. Journal of Sleep Research 2010;19:274.	
34	Emery PC, Wilson KG, Kowal J. Major depressive disorder and sleep disturbance in patients with chronic pain. Pain Res Manag. 2014; 19(1):35-41.	Wrong population: General chronic pain population.
35	Femia A, Lemaster C, Bertisch S, Saper R. Change in sleep quality in a randomized controlled trial of yoga, physical therapy, and education for low-income minorities with chronic low back pain. Journal of Alternative and Complementary Medicine 2016; 22(6):A74-A75.	Wrong publication type: Abstract, conference paper.
36	França VL, Koerich MH, Nunes GS. Sleep quality in patients with chronic low back pain. Fisioter. mov. 2015;28(4):803-810	Wrong outcome: No risk factors for insomnia are presented and there is insufficient data to calculate the odds ratios.
37	Gadiyar V, Mishra K: Incidence of sleep disturbance and secondary analgesics in patients attending chronic pain clinic. European Journal of Pain Supplements 2011; 5(1):177.	Wrong publication type: Abstract, conference paper.
38	Geneva-Popova M, Popova VS, Karalilova R, Popova SD. Insomnia in patients with chronic low back pain. Osteoporosis International 2017;28:S308-S309.	Wrong publication type: Abstract, conference paper.
39	Gerhart JI, Burns JW, Post KM, Smith DA, Porter LS, Burgess HJ, Schuster E, Buvanendran A, Fras AM, Keefe FJ. Relationships Between Sleep Quality and Pain-Related Factors for People with Chronic Low Back Pain: Tests of Reciprocal and Time of Day Effects. Ann Behav Med 2017; 51(3):365-375.	Wrong population: Inclusion of low back pain patients with specific diagnoses (e.g. degenerative disk disease, spinal stenosis, or disk herniation (radiculopathy subcategory)).
40	Hållstam A, Löfgren M, Benson L, Svensén C, Stålnacke BM. Assessment and treatment at a pain clinic: A one-year follow-up of patients with chronic pain. Scand J Pain. 2017; 17:233-242.	Wrong outcome: No risk factors for insomnia are presented and there is insufficient data to calculate the odds ratios.
41	Halonen JI, Shiri R, Hanson LLM, Lallukka T.<br>Risk and Prognostic Factors of Low Back Pain: Repeated Population-based Cohort Study in Sweden. Spine (Phila Pa 1976) 2019; 44(17):1248-1255.	Wrong outcome: No risk factors for insomnia are presented and there is insufficient data to calculate the odds ratios.

42	Harman K, Pivik RT, D'Eon JL, Wilson KG, Swenson JR, Matsunaga L. Sleep in depressed and nondepressed participants with chronic low back pain: electroencephalographic and behaviour findings. Sleep 2002; 25(7):775-783.	Wrong outcome: No risk factors for insomnia are presented and there is insufficient data to calculate the odds ratios.
43	Heffner KL, France CR, Trost Z, Ng HM, Pigeon WR. Chronic low back pain, sleep disturbance, and interleukin-6. Clin J Pain 2011; 27(1):35-41.	Wrong outcome: No risk factors for insomnia are presented and there is insufficient data to calculate the odds ratios.
44	Hong JH, Kim HD, Shin HH, Huh B. Assessment of depression, anxiety, sleep disturbance, and quality of life in patients with chronic low back pain in Korea. Korean J Anesthesiol. 2014; 66(6):444-450.	Wrong outcome: No risk factors for insomnia are presented and there is insufficient data to calculate the odds ratios.
45	Iglesias-González JJ, Muñoz-García MT, Rodrigues-de-Souza DP, Alburquerque-Sendín F, Fernández-de-las-Peñas C. Myofascial trigger points, pain, disability, and sleep quality in patients with chronic nonspecific low back pain. Pain Medicine 2013;14(12):1964-1970.	Wrong outcome: No risk factors for insomnia are presented and there is insufficient data to calculate the odds ratios.
46	Jungquist CR, O'Brien C, Matteson-Rusby S, Smith MT, Pigeon WR, Xia Y, Lu N, Perlis ML. The efficacy of cognitive-behavioral therapy for insomnia in patients with chronic pain. Sleep Med. 2010;11(3):302-309.	Wrong outcome: No risk factors for insomnia are presented and there is insufficient data to calculate the odds ratios.
47	Kanaan S, Siengsukon C, Arnold P, Burton D, Emmanuel N, Sharma N. Relationship between sleep quality and functional and psychological symptoms in patients with chronic low back pain. Physiotherapy (United Kingdom) 2015;101, supplement 1:eS713-eS714.	Wrong publication type: Abstract, conference paper
48	Kang JH, Chen HS, Chen SC, Jaw FS. Disability in patients with chronic neck pain: heart rate variability analysis and cluster analysis. Clin J Pain 2012; 28(9):797-803.	Wrong outcome: No risk factors for insomnia are presented and there is insufficient data to calculate the odds ratios.
49	Kang JH, Yeh JR, Chen SC. Morning pain exacerbation and sleep HRV in the chronic neck pain. International Journal of Rheumatic Diseases 2014; 17:116-117.	Wrong publication type: Abstract, conference paper.
50	Karaman S, Karaman T, Dogru S, Onder Y, Citil R, Bulut YE, Tapar H, Sahin A, Arici S, Kaya Z, Suren M. Prevalence of sleep disturbance in chronic pain. Eur Rev Med Pharmacol Sci. 2014; 18(17):2475-2481.	Wrong population: General chronic pain population.

51	Karkucak M, Ak E, Çapkin E. The relationship between the clinical findings and quality of life in female patients with chronic low back pain. Turkiye Fiziksel Tip ve Rehabilitasyon Dergisi 2013; 59(1):259.	Wrong publication type: Abstract, conference paper.
52	Kelly G, Blake C, Doody C, Burke ET, Power CK, Horan S, Mc Dermott P, Keeley V, Spencer O, Fullen BM. The impact of a cognitive behavioural therapy pain management programme on sleep and cognitive function: A pilot study. Physiotherapy (United Kingdom) 2011; 97:eS599	Wrong publication type: Abstract, conference paper.
53	Kim MG, Ahn YS. Associations Between Lower Back Pain and Job Types in South Korean Male Firefighters. Int J Occup Saf Ergon. 2019; 1-8.	Wrong population: Inclusion of low back pain patients with specific diagnoses (e.g. disc herniation and spinal stenosis).
54	Kim MG, Ahn YS. Associations Between Lower Back Pain and Job Types in South Korean Male Firefighters. Int J Occup Saf Ergon. 2019; 1-8.	Duplicate
55	Kim SA, Yang KI, Oh KY, Hwangbo Y. Association between sleep quality and myofascial pain syndrome in Korean adults: Questionnaire based study. Journal of Musculoskeletal Pain 2014; 22(3):232-236.	Wrong population: Participants were diagnosed with Myofascial Pain Syndrome. No subdivision were made regarding back, neck or spinal pain.
56	Kim SH, Sun JM, Yoon KB, Moon JH, An JR, Yoon DM. Risk factors associated with clinical insomnia in chronic low back pain: a retrospective analysis in a university hospital in Korea. Korean J Pain 2015; 28(2):137-143.	Wrong population: Inclusion of low back pain patients with specific diagnoses (e.g. Lumbar disc herniation, spinal stenosis, spondylolisthesis).
57	Kong X, Ferree T, Moynihan M, Gozani S. Effectiveness of fixed-site high-frequency transcutaneous electrical nerve stimulation among individuals with chronic pain and abnormal sleep. Postgraduate Medicine 2018; 130:89-91.	Wrong publication type: Abstract, conference paper.
58	Krokhmal S, Vyshlova I, Karpov S. New approach of correction anxiety, depression and sleep disturbances in patients with chronic non-specific lower back pain. Journal of the Neurological Sciences 2017; 381:978.	Wrong publication type: Abstract, conference paper.
59	Licciardone JC, Schmitt ME, Aryal S. Ostepathic and allopathic physician interpesonal manner, empathy, and communication style and clinical status of their patients: a pain registry-based study. J	Wrong outcome: No risk factors for insomnia are presented and there is insufficient data to calculate the odds ratios.

	Am Osteopath Assoc. 2019 [Epub ahead of print]	
60	Lin W, Burke L, Schlenk EA, Yeh CH. Use of an Ecological Momentary Assessment Application to Assess the Effects of Auricular Point Acupressure for Chronic Low Back Pain. Comput Inform Nurs. 2019; 37(5):276-282.	Wrong outcome: No risk factors for insomnia are presented and there is insufficient data to calculate the odds ratios.
61	Liszka-Hackzell JJ, Martin DP. Analysis of nighttime activity and daytime pain in patients with chronic back pain using a self-organizing map neural network. J Clin Monit Comput 2015; 19(6):411-414	Wrong outcome: No risk factors for insomnia are presented and there is insufficient data to calculate the odds ratios.
62	Maren EHN, Wang C, Mason ST. An apple a day: The impact of nutrition quality on severity of comorbid low back pain and insomnia. Psychosomatic Medicine 2013; 75(3):A-119	Wrong publication type: Abstract, conference paper.
63	Margarit C, Ballester P, Inda MDM, Roca R, Gomez L, Planelles B, Ajo R, Morales D, Peiro AM. OPRM1 Gene Interaction with Sleep in Chronic Pain Patients Treated with Opioids. Pain Physician 2019; 22(1):97-107.	Wrong outcome: No risk factors for insomnia are presented and there is insufficient data to calculate the odds ratios.
64	Marty M, Rozenberg S, Duplan B, Thomas P, Duquesnoy B, Allaert F. Quality of sleep in patients with chronic low back pain: a case-control study. Eur Spine J 2008; 17(6):839-844.	Wrong outcome: No risk factors for insomnia are presented and there is insufficient data to calculate the odds ratios.
65	Mateu M, Alda O, Inda M, Margarit C, Ajo R, Morales D, van-der Hofstadt CJ, Peiró AM. Randomized, Controlled, Crossover Study of Self-administered Jacobson Relaxation in Chronic, Nonspecific, Low-back Pain. Altern Ther Health Med. 2018;24(6):22-30.	Wrong outcome: No risk factors for insomnia are presented and there is insufficient data to calculate the odds ratios.
66	Mayer TG, Asih SR, Neblett R, Gatchel RJ. What is the responsiveness of clinical insomnia to interdisciplinary functional restoration in chronic disabling occupational spinal disorders? Spine Journal 2013; 13(9):15S.	Wrong publication type: Abstract, conference paper.
67	Miller K, Yarlas A, Wen W, Kowalski M, Lynch SY, Dain B, Ripa SR. Correlates of improvement in physical quality of life and quality of sleep among chronic low back pain patients with treatment with buprenorphine transdermal system (BTDS) Value in Health – 2013; 16(3):A122.	Wrong publication type: Abstract, conference paper.
68	Minetto MA, Gamerro G, Gays G, Vigo S, Caresio C, Gorji NE, Massazza G.	Wrong outcome: No risk factors for insomnia are

	Effectiveness of an Innovative Mattress Overlay for Improving Rehabilitation in Low Back Pain: A Pilot Randomized Controlled Study. J Back Musculoskelet Rehabil. 2018; 31(6):1075-1083.	presented and there is insufficient data to calculate the odds ratios.
69	Minetto MA, Gamerro G, Gays G, Vigo S, Caresio C, Gorji NE, Massazza G. Effectiveness of an Innovative Mattress Overlay for Improving Rehabilitation in Low Back Pain: A Pilot Randomized Controlled Study. J Back Musculoskelet Rehabil. 2018; 31(6):1075-1083.	Duplicate
70	Miranda H, Viikari-Juntura E, Punnett L, Riihimaeki H. Age modifies the risk of low back pain related to occupational loading, health behaviours, sleep disturbance and mental stress. European Journal of Public Health 2007; 17(suppl_2): 55.	Wrong publication type: Abstract, conference paper.
71	Moloney N, Beales D, Azoory R, Hübscher M, Waller R, Gibbons R, Rebbeck T. Are measures of pain sensitivity associated with pain and disability at 12-month follow up in chronic neck pain? Musculoskeletal Care. 2018; 16(4):415-424.	Wrong outcome: No risk factors for insomnia are presented and there is insufficient data to calculate the odds ratios.
72	Moloney N, Beales D, Azoory R, Hübscher M, Waller R, Gibbons R, Rebbeck T. Are measures of pain sensitivity associated with pain and disability at 12-month follow up in chronic neck pain? Musculoskeletal Care. 2018; 16(4):415-424.	Duplicate
73	Morelhao PK, Kim LJ, Pinto RZ, Tufik S, Andersen ML. Should Physical Therapists Assess Sleep Quality in Patients Seeking Care for Low Back Pain? Phys Ther. 2019; 99(8):961-963.	Wrong outcome: No risk factors for insomnia are presented and there is insufficient data to calculate the odds ratios.
74	Muñoz-Muñoz S, Muñoz-García MT, Alburquerque-Sendín F, Arroyo-Morales M, Fernández-De-Las-Peñas C. Myofascial trigger points, pain, disability, and sleep quality in individuals with mechanical neck pain. Journal of Manipulative and Physiological Therapeutics 2012; 35(8): 608-613.	Wrong outcome: No risk factors for insomnia are presented and there is insufficient data to calculate the odds ratios.
75	Murase K, Tabara Y, Ito H, Kobayashi M, Takahashi Y, Setoh K, Kawaguchi T, Muro S, Kadotani H, Kosugi S, Sekine A, Yamada R, Nakayama T, Mishima M, Matsuda S, Matsuda F, Chin K. Knee Pain and Low Back Pain Additively Disturb Sleep in the General Population: A Cross-Sectional Analysis of the	Wrong outcome: No risk factors for insomnia are presented and there is insufficient data to calculate the odds ratios.

	Nagahama Study. PLoS One 2015; 10(10):e0140058.	
76	Murphy SL, Harris RE, Keshavarzi NR, Zick SM. Self-Administered Acupressure for Chronic Low Back Pain: A Randomized Controlled Pilot Trial. Pain Med. 2019; 20(12):2588-2597.	Wrong outcome: No risk factors for insomnia are presented and there is insufficient data to calculate the odds ratios.
77	Musich S, Wang SS, Slindee L, Kraemer S, Yeh CS. Association of Resilience and Social Networks With Pain Outcomes Among Older Adults. Popul Health Manag. 2019; 22(6):511-521.	Wrong population: Sample of mixed pain population including back pain, osteoarthritis and rheumatoid arthritis.
78	Nandini B, Mooventhan A, Manjunath NK. Add-on Effect Of Hot Sand Fomentation To Yoga On Pain, Disability, And Quality Of Life In Chronic Neck Pain Patients. Explore (NY). 2018; 14(5):373-378.	Wrong outcome: No risk factors for insomnia are presented and there is insufficient data to calculate the odds ratios.
79	O'Brien EM, Waxenberg LB, Atchison JW, Gremillion HA, Staud RM, McCrae CS, Robinson ME. Negative mood mediates the effect of poor sleep on pain among chronic pain patients. Clin J Pain 2010; 26(4):310-319.	Wrong outcome: No risk factors for insomnia are presented and there is insufficient data to calculate the odds ratios.
80	O'Donoghue GM, Fox N, Heneghan C, Hurley DA. Objective and subjective assessment of sleep in chronic low back pain patients compared with healthy age and gender matched controls: a pilot study. 2009; 10:122	Wrong outcome: No risk factors for insomnia are presented and there is insufficient data to calculate the odds ratios.
81	Oh JS, Lee KS. Relationship of Pain and Anger in Patients with Back or Neck Pain. Kor J Spine 2009; 6(1):22-26.	Foreign language
82	Owens M, Bulls H, Gossett E, Wesson-Sides K, Terry S, Goodin B. Sleep disturbance in chronic low back pain: An age- and gender-matched case-control study. Journal of Pain 2015; 16(4): S29.	Wrong publication type: Abstract, conference paper.
83	Paanalahti K, Wertli MM, Held U, Akerstedt T, Holm LW, Nordin M, Skillgate E. Spinal pain-good sleep matters: a secondary analysis of a randomized controlled trial. European Spine Journal 2015; 25(3):760-765.	Wrong population: Subjects with non-specific pain and disability in the back and/or neck lasting for at least 2 weeks were eligible.
84	Pakpour AH, Yaghoubidoust M, Campbell P. Persistent and Developing Sleep Problems: A Prospective Cohort Study on the Relationship to Poor Outcome in Patients Attending a Pain Clinic with Chronic Low Back Pain. Pain Pract 2018; 18(1):79-86.	Wrong outcome: No risk factors for insomnia are presented and there is insufficient data to calculate the odds ratios.
85	Pakpour AH, Yaghoubidoust M, Campbell P. Persistent and Developing Sleep Problems: A	Duplicate

	Prospective Cohort Study on the Relationship to Poor Outcome in Patients Attending a Pain Clinic with Chronic Low Back Pain. Pain Pract 2018; 18(1):79-86.	
86	Pinheiro MB, Morosoli JJ, Ferreira ML, Madrid-Valero JJ, Refshauge K, Ferreira PH, Ordonana JR. Genetic and Environmental Contributions to Sleep Quality and Low Back Pain: A Population-Based Twin Study. Psychosom Med. 2018; 80(3):263-270.	Wrong outcome: No risk factors for insomnia are presented and there is insufficient data to calculate the odds ratios.
87	Pinheiro MB, Morosoli JJ, Ferreira ML, Madrid-Valero JJ, Refshauge K, Ferreira PH, Ordonana JR. Genetic and Environmental Contributions to Sleep Quality and Low Back Pain: A Population-Based Twin Study. Psychosom Med. 2018; 80(3):263-270.	Duplicate
88	Purushothaman B, Singh A, Lingutla K, Bhatia C, Pollock R, Krishna M. Prevalence of insomnia in patients with chronic back pain. J Orthop Surg (Hong Kong) 2013; 21(1):68-70.	Wrong outcome: No risk factors for insomnia are presented and there is insufficient data to calculate the odds ratios.
89	Rhon DI, O'Hagan E, Mysliwiec V, Lentz TA. Does Disordered Sleep Moderate the Relationship Between Pain, Disability and Downstream Health Care Utilization in Patients With Low Back Pain?: A Longitudinal Cohort From the US Military Health System. Spine (Phila Pa 1976). 2019; 44(21):1481-1491.	Wrong outcome: No risk factors for insomnia are presented and there is insufficient data to calculate the odds ratios.
90	Robertson JA, Purple RJ, Cole P, Zaiwalla Z, Wulff K, Pattinson KT. Sleep disturbance in patients taking opioid medication for chronic back pain. Anaesthesia 2016; 71(11):1296-1307.	Wrong outcome: No risk factors for insomnia are presented and there is insufficient data to calculate the odds ratios.
91	Rodrigues-De-Souza DP, Fernández-De-Las-Peñas C, Martín-Vallejo FJ, Blanco-Blanco JF, Moro-Gutiérrez L, Alburquerque-Sendín F. Differences in pain perception, health-related quality of life, disability, mood, and sleep between Brazilian and Spanish people with chronic non-specific low back pain. Braz J Phys Ther 2016; 20(5):412-421.	Wrong outcome: No risk factors for insomnia are presented and there is insufficient data to calculate the odds ratios.
92	Ropponen A, Silventoinen K, Hublin C, Svedberg P, Koskenvuo M, Kaprio J. Sleep patterns as predictors for disability pension due to low back diagnoses: a 23-year longitudinal study of Finnish twins. Sleep 2013; 36(6):891-897.	Wrong outcome: No risk factors for insomnia are presented and there is insufficient data to calculate the odds ratios.
93	Schlesinger I, Hering-Hanit R, Dagan Y. Sleep disturbances after whiplash injury:	Wrong outcome: No risk factors for insomnia are

	objective and subjective findings. Headache 2001; 41(6): 586-589.	presented and there is insufficient data to calculate the odds ratios.
94	Şekeröz S, Öztop M, Aslan Telci E, Yağci N, Tekin F. The effect of severe neck pain on sleep and quality of life in university students. Fiz. Rehab. 2017; 28(2):S89.	Wrong publication type: Abstract, conference paper.
95	Sezgin M, Hasanefendioglu EZ, Sungur MA, Incel NA, Cimen OB, Kanik A, Sahin G. Sleep quality in patients with chronic low back pain: a cross-sectional study assesing its relations with pain, functional status and quality of life. J Back Musculoskelet Rehabil 2015; 28(3):433-41.	Wrong outcome: No risk factors for insomnia are presented and there is insufficient data to calculate the odds ratios.
96	Shyen S, Amine B, Binoune I, Gueddari S, Hajjaj-Hassouni N. The prevalence of sleep disorders and their determinants in patients with chronic neck pain. Ann Rheum Dis 2014; 73(Suppl2):305.	Wrong publication type: Abstract, conference paper.
97	Sharma S, Jensen MP, Moseley GL, Abbott JH. Results of a Feasibility Randomised Clinical Trial on Pain Education for Low Back Pain in Nepal: The Pain Education in Nepal-Low Back Pain (PEN-LBP) Feasibility Trial. BMJ Open. 2019 Mar 27;9(3):e026874.	Wrong outcome: No risk factors for insomnia are presented and there is insufficient data to calculate the odds ratios.
98	Slavish D, Graham-Engel JE, Martire L, Smyth J. Bidirectional associations between daily pain, affect, and sleep quality in young adults with and without chronic back pain. Journal of Pain 2017; 18(4):S73.	Wrong publication type: Abstract, conference paper.
99	Sorensen G, Stoddard AM, Stoffel S, Buxton O, Sembajwe G, Hashimoto D, Dennerlein JT, Hopcia K. The role of the work context in multiple wellness outcomes for hospital patient care workers. J Occup Environ Med 2011; 53(8):899-910.	Wrong population: About 2/3rd of the sample was a shift worker.
100	Soysal M, Kara B, Arda MN. Assessment of Physical Activity in Patients with Chronic Low Back or Neck Pain. Turkish Neurosurgery 2013; 23(1): 75-80.	Wrong outcome: No risk factors for insomnia are presented and there is insufficient data to calculate the odds ratios.
101	Sribastav SS, He P, Long J, Li Z, Wei F, Wang J, Liu H, Wang H, Zheng Z. Interplay among pain intensity, sleep disturbance and emotion in patients with non-specific low back pain. PeerJ 2017; 5:e3282.	Wrong population: Uncertain if all participants had chronic low back pain since people with low back pain which last more than one month were eligible.
102	Srivastava S, Yadav P, Panchal BN,Vala AU, Ratnani I, Khania P. Association of depression and chronic lower-back pain.	Wrong population: Inclusion of patients with low back pain with different (specific)

	Archives of Psychiatry and Psychotherapy, 2018; 4:37–46.	etiologies (trauma, tumor, others).
103	Srivastava S, Yadav P, Panchal BN,Vala AU, Ratnani I, Khania P. Association of depression and chronic lower-back pain. Archives of Psychiatry and Psychotherapy, 2018; 4:37–46.	Duplicate
104	Stanisławska I, Mincewicz M, Cabak A, Kaczor R, Czarny-Działak M, Witek B, Łyp M. Epidemiological Aspects of Low Back Pain. Adv Exp Med Biol. 2019;1176:47-52.	Wrong outcome: No risk factors for insomnia are presented and there is insufficient data to calculate the odds ratios.
105	Sturgeon J, Middleton S, Rico T, Mackey S, ; K. Johnson. Differential daily effects of pain intensity, sleep, and mood on physical activity in chronic back pain. Journal of Pain 2016; 17(4):S16.	Wrong publication type: Abstract, conference paper.
106	Sturgeon JA, Hah JM, Sharifzadeh Y, Middleton SK, Rico T, Johnson KA, Mackey SC. Predictors of Daily Pain Medication Use in Individuals With Recurrent Back Pain. Int J Behav Med 2018; 25(2):252-258.	Wrong outcome: No risk factors for insomnia are presented and there is insufficient data to calculate the odds ratios.
107	Taguchi T, Nozawa K, Parsons B, Yoshiyama T, Ebata N, Igarashis A, Fujii K. Effectiveness of pregabalin for treatment of chronic cervical radiculopathy with upper limb radiating pain: an 8-week, multicenter prospective observational study in Japanese primary care settings. J Pain Res. 2019; 12:1411-1424.	Wrong outcome: No risk factors for insomnia are presented and there is insufficient data to calculate the odds ratios.
108	Taylor KA, Schwartz SW. The association between poor sleep and chronic low back pain: An analysis of 2012 national health interview survey data. Sleep 2018; 41(suppl_1):A336-A337.	Wrong publication type: Abstract, conference paper.
109	Thong ISK, Tan G, Jensen MP. The buffering role of positive affect on the association between pain intensity and pain related outcomes. Scand J Pain. 2017; 14:91-97.	Wrong population: Mixed sample of patients with the diagnosis chronic back pain or chronic knee pain.
110	Tompkins D, Martel M, Smith M, Campbell C, Edwards R. Does insomnia modify the effect of catastrophizing on psychophysical pain testing in patients with chronic pain? Journal of Pain 2014; 15(4):S22.	Wrong publication type: Abstract, conference paper.
111	Uchmanowicz I, Koltuniuk A, Stepien a, Uchmanowicz B, Rosinczuk J. The influence of sleep disorders on the quality of life in patients with chronic low back pain. Scandinavian Journal of Caring Sciences 2019; 33(1):119-127.	Wrong outcome: No risk factors for insomnia are presented and there is insufficient data to calculate the odds ratios.

112	Uchmanowicz I, Koltuniuk A, Stepien a, Uchmanowicz B, Rosinczuk J. The influence of sleep disorders on the quality of life in patients with chronic low back pain. Scandinavian Journal of Caring Sciences 2019; 33(1):119-127.	Duplicate
113	Valenza MC, Valenza G, González-Jiménez E, De-la-Llave-Rincón AI, Arroyo-Morales M, Fernández-de-Las-Peñas C. Alteration in Sleep Quality in Patients With Mechanical Insidious Neck Pain and Whiplash-Associated Neck Pain. Am J Phys Med Rehabil. 2012; 91(7):584-91.	Wrong outcome: No risk factors for insomnia are presented and there is insufficient data to calculate the odds ratios.
114	van de Water AT, Eadie J, Hurley DA. Investigation of sleep disturbance in chronic low back pain: an age- and gender-matched case-control study over a 7-night period. Man Ther 2011; 16(6):550-556.	Wrong outcome: No risk factors for insomnia are presented and there is insufficient data to calculate the odds ratios.
115	van de Water AT, Eadie J, Hurley DA. Sleep patterns in people with chronic low back pain compared to an age and gender matched control group over a 7-night period. Journal of Sleep Research 2010; 19:270-271.	Wrong publication type: Abstract, conference paper.
116	Vanti C, Banchelli F, Marino C, Puccetti A, Guccione AA, Pillastrini P. Effectiveness of a "Spring Pillow" Versus Education in Chronic Nonspecific Neck Pain: A Randomized, Controlled Trial. Phys Ther. 2019; 99(9):1177-1188.	Wrong outcome: No risk factors for insomnia are presented and there is insufficient data to calculate the odds ratios.
117	Vega RDL, Miró J, Esteve R, Ramírez-Maestre C, López-Martínez AE, Jensen MP. Sleep disturbance in individuals with physical disabilities and chronic pain: The role of physical, emotional and cognitive factors. Disability and Health Journal 2019; 12(4):588-593.	Wrong population: Mixed sample including patients with spinal cord injury, multiple sclerosis, back pain, osteoarthritis , amputation or limb loss, diabetes, postpolio syndrome and muscular dystrophy.
118	Yang H, Haldeman S. Behavior-Related Factors Associated With Low Back Pain in the US Adult Population. Spine (Phila Pa 1976) 2018; 43(1):28-34.	Wrong outcome: No risk factors for insomnia are presented and there is insufficient data to calculate the odds ratios.
119	Yeh CH, Suen LKP, Shen J, Chien LC, Liang Z, Glick RM, Morone NE, Chasens ER. Changes in Sleep With Auricular Point Acupressure for Chronic Low Back Pain. Behavioral Sleep Medicine 2016; 14(3):279-294.	Wrong outcome: No risk factors for insomnia are presented and there is insufficient data to calculate the odds ratios.
120	Yoshimoto T, Oka H, Fujii T, Kawamata K, Kokaze A, Koyama Y, Matsudaira K. Survey	Wrong outcome: No risk factors for insomnia are

	on Chronic Disabling Low Back Pain Among Care Workers at Nursing Care Facilities: A Multicenter Collaborative Cross-Sectional Study. J Pain Res. 2019;12:1025-1032.	presented and there is insufficient data to calculate the odds ratios.
121	Yoshimoto T, Oka H, Fujii T, Kawamata K, Kokaze A, Koyama Y, Matsudaira K. Survey on Chronic Disabling Low Back Pain Among Care Workers at Nursing Care Facilities: A Multicenter Collaborative Cross-Sectional Study. J Pain Res. 2019;12:1025-1032.	Duplicate
122	Zarrabian MM, Johnson M, Kriellaars D. Relationship Between Sleep, Pain, and Disability in Patients With Spinal Pathology. Arch Phys Med Rehabil. 2014;95(8):1504-1509.	Wrong population: Inclusion of low back pain patients with specific diagnoses (e.g. stenosis, disc herniation, remote compression fracture).
