# Supplementary material for: Associates of Insomnia in People with Chronic Spinal Pain: A Systematic Review and Meta-Analysis
Source: J Clin Med. 2021 Jul 19;10(14):3175. doi: 10.3390/jcm10143175 (PMC8304652; doi:10.3390/jcm10143175)
Supplement: Supplementary file 1 [file jcm-10-03175-s001.zip › Table S3 Quality of Evidence.pdf]

Table S3. *Quality of Evidence - Modified version of the Grading of Recommendations Assessment, Development, and Evaluation criteria*

Quality assessment									
Factor meta-analysis	Downgraded						Upgraded		
	Phase of investigation[1]	Study limitations[2]	Inconsistency[3]	Indirectness[4]	Imprecision[5]	Publication bias[6]	Effect size[7]	Exposure -response gradient	Overall
Sex	↓					↓[#]			Low
Age	↓					↓[#]			Low
Body mass index	↓					↓[#]			Low
Physical activity	↓		↓	↓		↓[#]			Very Low
Subgroup analysis – physical activity	↓					↓[#]			Low
Professional activity	↓					↓[#]			Low
Comorbidities	↓		↓			↓[#]			Very Low
Pain intensity	↓					↓[#]	↑		Moderate
Depression	↓					↓[#]	↑		Moderate
Anxiety	↓					↓[#]	↑		Moderate

[1] Cross-sectional studies
[2] > 25% of the participants from studies with high risk of bias
[3] Heterogeneity was based on similarity of point estimates, extent of overlap of confidence intervals, and I^2 test (> 50%).
[4] Indirectness: > 25% of results from that failure to apply appropriate eligibility criteria and had poor measurement of both exposure and outcome.
[5] Fewer than 400 participants in the pooling
[6] Funnel plot and Egger's test
[7] Odds ratio > 2.5
↑ Upgraded
↓ Downgraded
[#] Unclear: Unable to assess publication bias (< 10 studies)
